# Analysis of chemical exchange saturation transfer contributions from brain metabolites to the Z-spectra at various field strengths and pH

**DOI:** 10.1038/s41598-018-37295-y

**Published:** 2019-01-31

**Authors:** Vitaliy Khlebnikov, Wybe J. M. van der Kemp, Hans Hoogduin, Dennis W. J. Klomp, Jeanine J. Prompers

**Affiliations:** Department of Radiology, Image Sciences Institute, University Medical Center Utrecht, Utrecht, The Netherlands

## Abstract

Chemical exchange saturation transfer (CEST) exploits the chemical exchange of labile protons of an endogenous or exogenous compound with water to image the former indirectly through the water signal. Z-spectra of the brain have traditionally been analyzed for two most common saturation phenomena: downfield amide proton transfer (APT) and upfield nuclear Overhauser enhancement (NOE). However, a great body of brain metabolites, many of interest in neurology and oncology, contributes to the downfield saturation in Z-spectra. The extraction of these “hidden” metabolites from Z-spectra requires careful design of CEST sequences and data processing models, which is only possible by first obtaining CEST signatures of the brain metabolites possessing labile protons. In this work, we measured exchange rates of all major-for-CEST brain metabolites in the physiological pH range at 37 °C. Analysis of their contributions to Z-spectra revealed that regardless of the main magnetic field strength and pH, five main contributors, i.e. myo-inositol, creatine, phosphocreatine, glutamate, and mobile (poly)peptides, account for ca. 90% of downfield CEST effect. The fundamental CEST parameters presented in this study can be exploited in the design of novel CEST sequences and Z-spectra processing models, which will enable simultaneous and quantitative CEST imaging of multiple metabolites: multicolor CEST.

## Introduction

Proton (^1^H)-magnetic resonance spectroscopy (MRS) is an established molecular profiling technique used in many research centers worldwide for pathological assessments. The large voxel size of MRS, however, is a limitation when it comes to metabolic profiling of highly heterogeneous diseased tissues such as tumorous tissue.

Complementary to ^1^H-MRS for metabolic imaging is Chemical Exchange Saturation Transfer (CEST)-magnetic resonance imaging (MRI). CEST is an imaging technique, which exploits chemical exchange of labile protons of a particular (endogenous or exogenous) CEST agent with water to detect the former indirectly with high sensitivity through the water signal^1^. In CEST, a Z-spectrum, which is a plot of the water signal attenuation versus off-resonance saturation frequency (Δω, Δω = 0 for water resonance), is normally acquired. The labile protons of endogenous tissue metabolites contribute to the water signal attenuation downfield (Δω = 0–5 ppm) from the water resonance. CEST has been exploited in metabolite-weighted imaging of glutamate^2^, glucose^3,4^, glycogen^5^, creatine^6^, myo-inositol^7^, glycosaminoglycans^8^, and mobile proteins and peptides rich in amide protons (these metabolites are abbreviated in this work as “mobile amides”)^9^. Thus far, the measurement efforts have mainly resulted in single metabolite-weighted (<75% purity) contrast, e.g. for glutamate^2^. Still, contamination of the contrast with other metabolites (other 25% or more) may result in misinterpretation of glutamate-CEST contrast in pathology. Ideally, one would like to use optimized CEST sequences for simultaneous imaging of multiple metabolites and decompose the frequency-dependent metabolite-weighted contrast in the Z-spectra into corresponding (multicolor) metabolic maps. However, the design of multicolor CEST sequences and advanced data processing models requires a precise knowledge of metabolic CEST signatures in the tissue of interest.

Multiple methods have been implemented for the extraction of downfield amide proton transfer (APT) and upfield nuclear Overhauser enhancement (NOE)^10–13^, two dominant saturation effects in brain Z-spectra^10,11,14^. The extraction of other clinically relevant “hidden” metabolites, such as those mentioned above, from brain Z-spectra is a major challenge, as it is still unknown in which proportions the exchangeable protons of the different brain metabolites contribute to the Z-spectra and how their contribution depends on field strength, pH and sequence parameters.

The aim of this work was to analyze CEST contributions from brain metabolites to the Z-spectra at different field strengths and pH. To this end, the exchange rate, arguably the most important parameter for CEST experiments, of major brain metabolites was measured at 14.1T in the physiological pH range at 37 °C. Subsequently, the CEST signatures of those metabolites were used in Bloch-McConnell equation (BME)^15^ simulations to find the main contributors to brain Z-spectra at different field strengths and pH as a function of saturation frequency offset. This in turn was used to optimize CEST-prepulse parameters for metabolic imaging at different field strengths.

The information on CEST-relevant metabolic parameters and CEST-prepulse parameters provided in this study, can be exploited in the design of novel CEST sequences and advanced Z-spectra processing models, which is the first step towards simultaneous and quantitative CEST imaging of multiple metabolites on par with MRS: multicolor CEST^16^.

## Results

### Major brain metabolites and their characteristics

**Figure 1:**
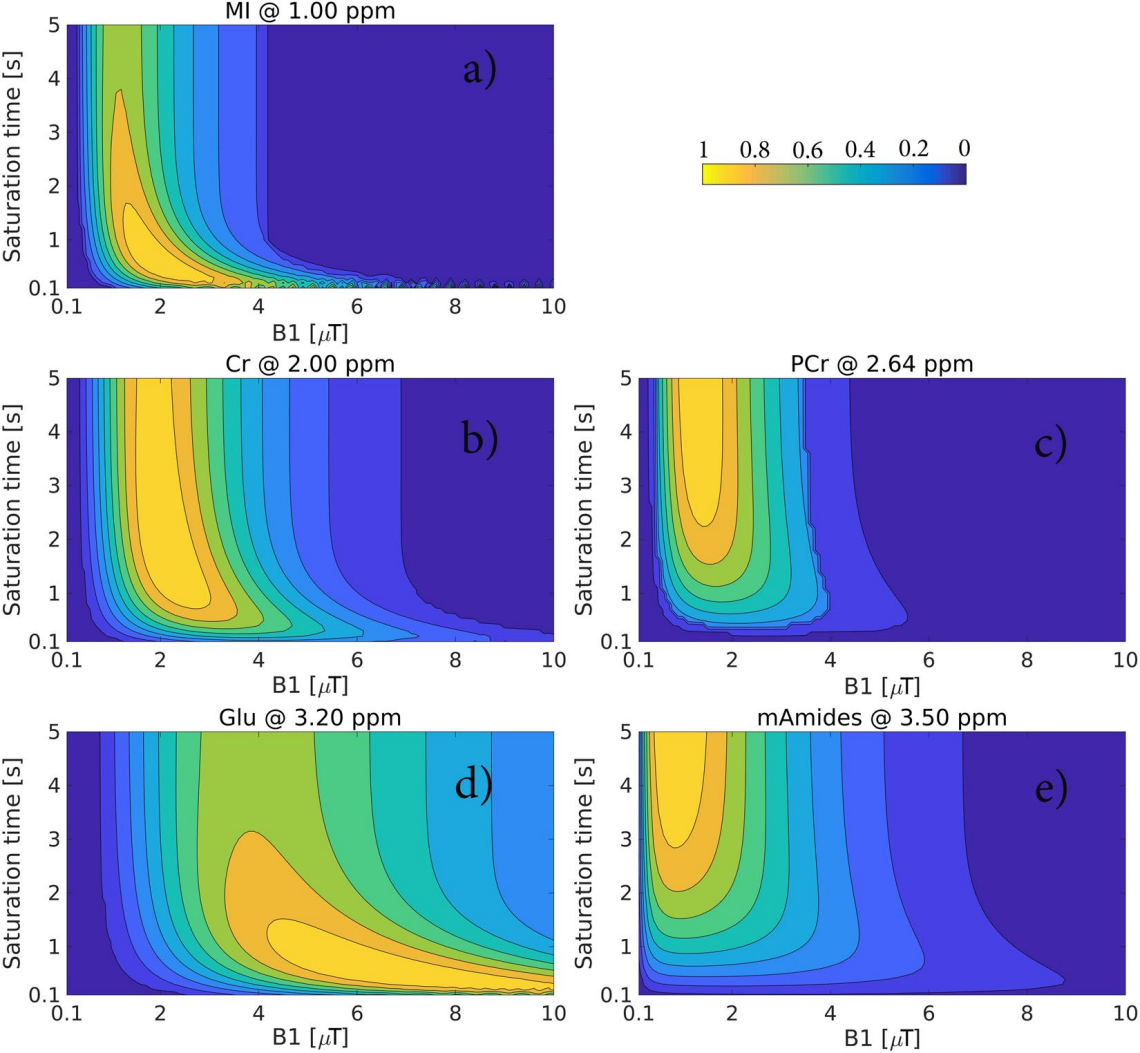
BME-simulated normalized effect size (pH = 7, 7 T field strength) for the following metabolites at their resonance frequencies: (**a**) MI (at 1.00 ppm), (**b**) Cr (at 2.00 ppm), (**c**) PCr (at 2.64 ppm), (**d**) Glu (at 3.20 ppm) and (**e**) mAmides (at 3.50 ppm) as a function of B1 amplitude and saturation time. Each map is an average of normalized (by maximum) maps simulated with WM and GM water T1 and T2 relaxation times. The contour plots overlaid on the maps delineate the regions with the effect size variation within 10%. See top right corner for color coding legend.

**Figure 2:**
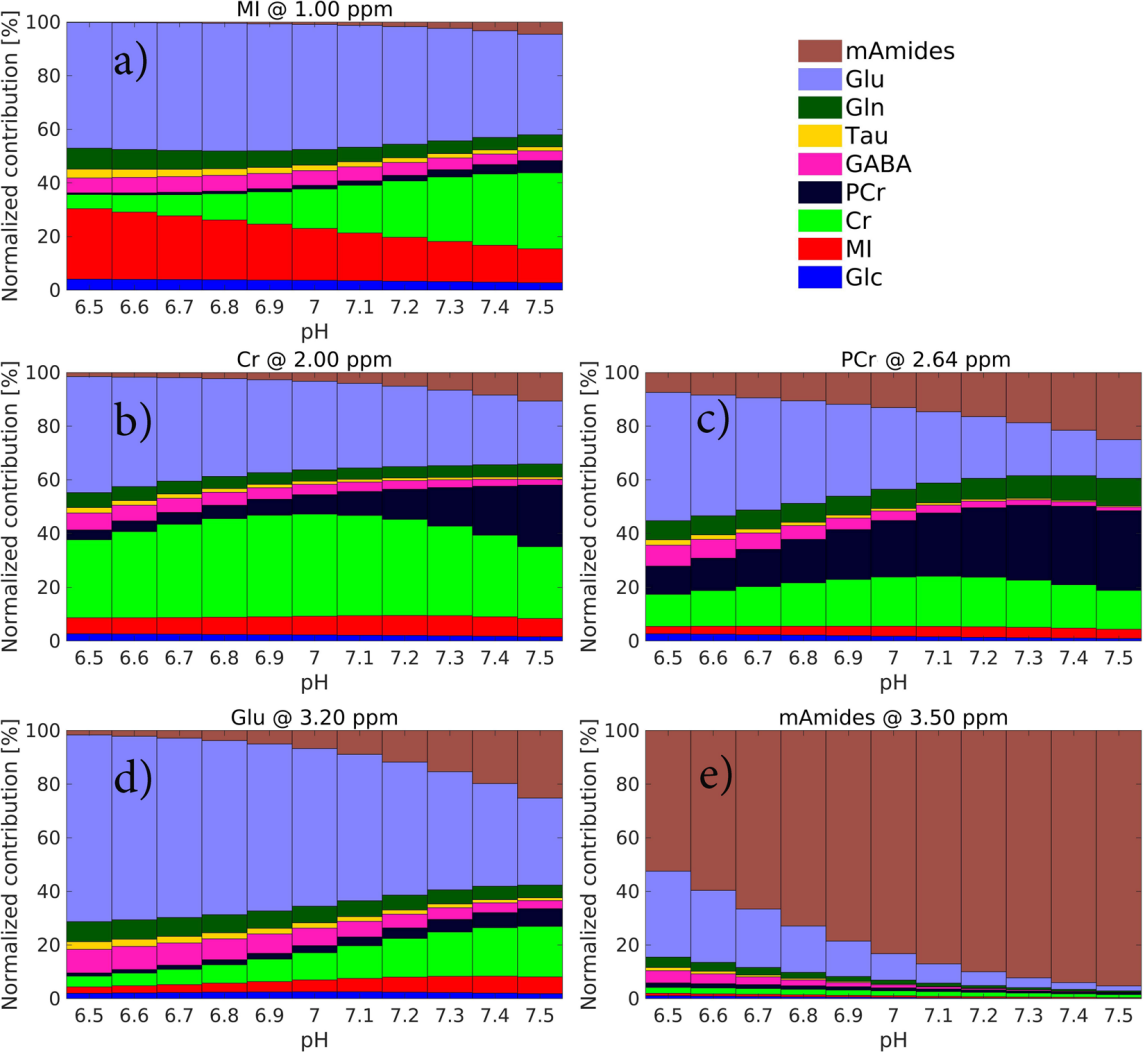
Stacked bar plots, of BME-simulated data for 7 T field strength, showing metabolic contributions to the Z-spectra in GM at the resonance frequency of: (**a**) MI (at 1.00 ppm), (**b**) Cr (at 2.00 ppm), (**c**) PCr (at 2.64 ppm), (**d**) Glu (at 3.20 ppm) and (**e**) mAmides (at 3.50 ppm) as a function of pH. The CEST-prepulse parameters yielding the largest CEST effects were determined at pH = 7 (see Table 2) and then applied to other pH levels. See top right corner for color coding legend.

**Figure 3:**
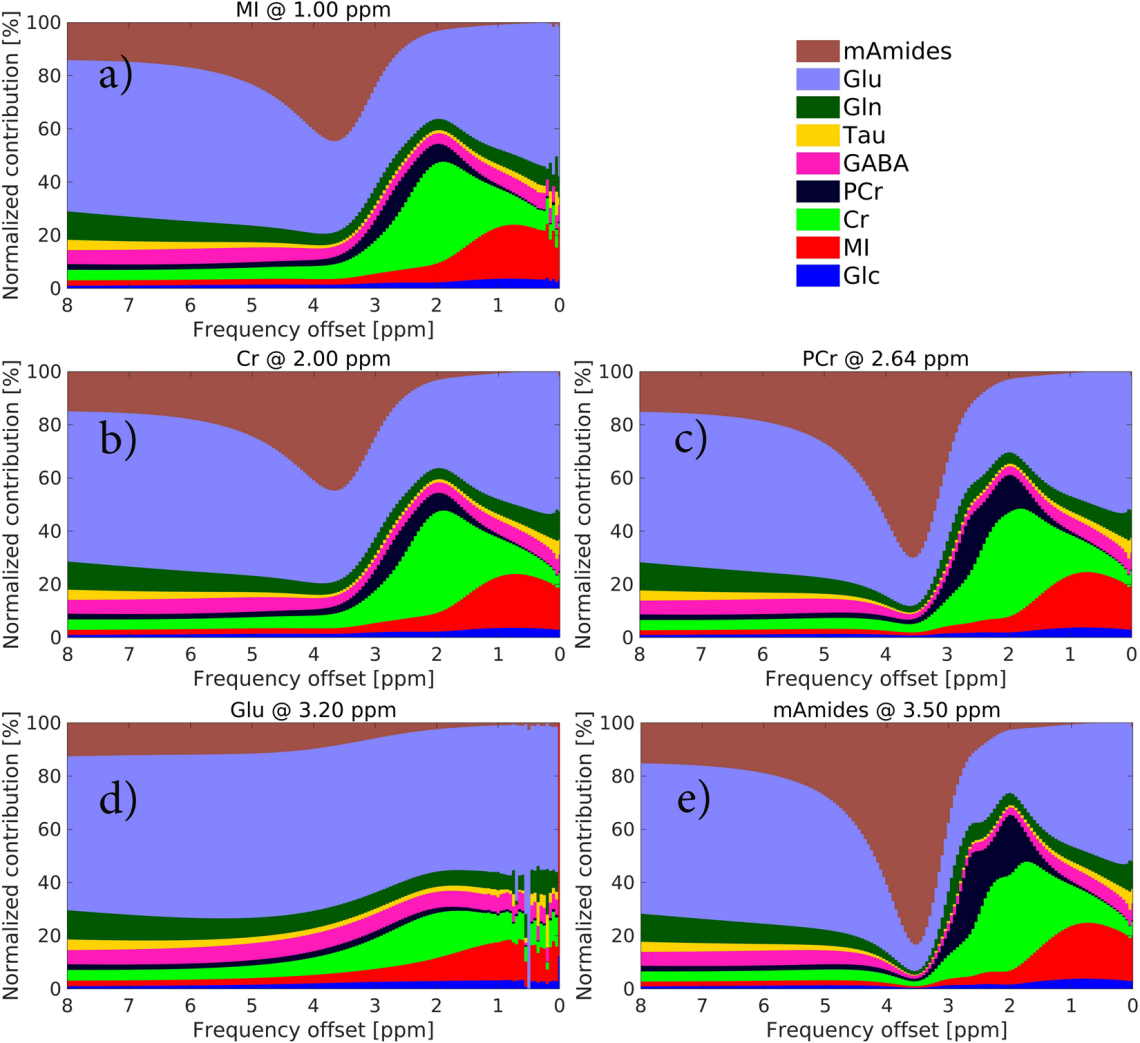
Stacked bar plots, of BME-simulated data (pH = 7, 7T field strength), showing metabolic contributions to the Z-spectra in GM as a function of frequency offset. Each subplot was simulated with the saturation scheme yielding the largest CEST effect (see Table 2) for: (**a**) MI, (**b**) Cr, (**c**) PCr, (**d**) Glu and (**e**) mAmides. See top right corner for color coding legend.

**Figure 4:**
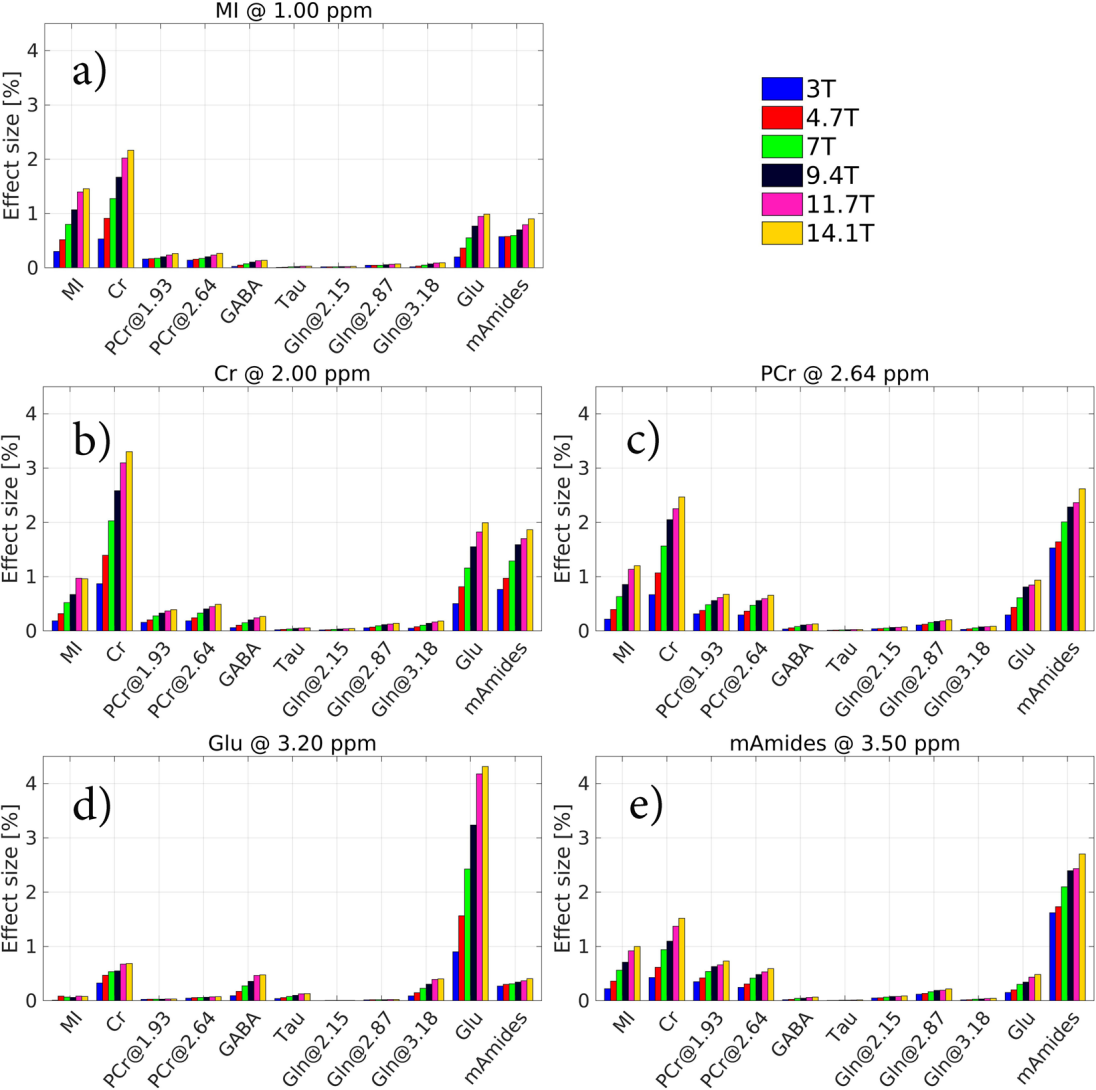
The comparison of BME-simulated CEST effects (pH = 7) from the brain metabolites in GM (see Table 1) at different field strengths. Shown are CEST effects from the individual exchangeable protons (X-axis, see Table 1 for their resonance frequencies). Each subplot was simulated at the CEST parameters yielding the largest CEST effect (see Table 2) for each particular metabolite at its resonance frequency: (**a**) MI (at 1.00 ppm), (**b**) Cr (at 2.00 ppm), (**c**) PCr (2.64 ppm), (**d**) Glu (3.20 ppm) and (**e**) mAmides (3.50 ppm). See top right corner for color coding legend. See Figs 26S–27S in Supplementary Material for Glc CEST effects.

The major metabolites as a source of CEST contrast in the brain were identified from^17^ taking into account their *in vivo* concentrations, and type and number of exchangeable protons. Metabolic solutions in 10 mM phosphate-buffered saline with different pH values (6.4, 6.7, 7.0 and 7.3) were prepared for all of the identified metabolites. For each metabolite at each pH value, Z-spectra were acquired at multiple B1 saturation levels of the CEST-prepulse on a 600 MHz NMR spectrometer at 37 °C. To determine the resonance frequencies, exchange rates and T2 relaxation times of labile protons, full Bloch-McConnell equations (BME) were fitted to the Z-spectra of the *in vitro* metabolic solutions. Those experimental results at a pH of 7.0 (typical intracellular pH^18^) are summarized in Table 1, which also contains the literature values for the exchange rates and *in vivo* metabolic concentrations in the brain. It is interesting to note that the error in the fitted resonance frequencies was less than 0.01 ppm (95% confidence interval). Considering that a single resonance frequency was fitted at all pH levels, such a small error suggests that pH dependence of the frequency offsets (metabolites with respect to the water resonance) in the studied pH range of 6.4–7.3 is negligible. The Z-spectra with BME fits (Figs 1S–9S) and 600 MHz ^1^H-NMR spectra (Figs 10S–18S) for all metabolites are provided in the Supplementary Material.

**Table 1:** The characteristics of the labile protons of major brain metabolites at 37 °C.

Metabolites (major species at pH = 7)	k0 and kb are the exchange rate constants (Hz) due to the spontaneous, and base catalysis. (Numbering means proton assignment)	T2 (ms)	*k* (Hz) pH = 7 (this work)	*k* (Hz) pH = 7 (liter.)	Labile protons (N)	*In-vivo* brain concentration (mM)
D-Glucose (Glc)	1 (42.7±1.9% α-isomer @ 2.18 ppm) k0 = 640, kb = 2.90e + 10	6.9 ± 2.7	3860 ± 110	—	1	1.0^17,50,51^
	1 (57.3 ± 1.9% β-isomer @ 2.88 ppm) k0 = 1470, kb = 2.89e + 10		4750 ± 90	—	1	
	2 (O_2_H @ 1.10 ppm) k0 = 0, kb = 3.61e + 10		3940 ± 260	—	1	
	3 and 4 (O_3,4_H @ 1.39 ppm) k0 = 820, kb = 1.73e + 10		2560 ± 40	—	2	
	5 (O_6_H @ 0.74 ppm) k0 = 530, kb = 4.91e + 09		950 ± 55	—	1	
Myo-Inositol (MI)	(@ 1.00 ppm) k0 = 760, kb = 1.39e + 10	22.8 ± 7.7	2090 ± 100	600^7^ pH=7.4	6	4.9/5.9^50,52^ WM/GM
Creatine (Cr)	(@ 2.00 ppm) k0 = 0, kb = 7.81e + 09	7.1 ± 0.2	810 ± 14	950^33^ ± 100	4	tCr^50,52–54^ 5.7/8.4 WM/GM
Phosphocreatine (PCr)	1 (@ 1.93 ppm) k0 = 26, kb = 4.21e + 08	7.8 ± 0.4	67 ± 8	120^33^ ±50	2	For brain^17^ Cr/Pcr≈1/1
	2 (@ 2.64 ppm) k0 = 11, kb = 1.17e + 09		126 ± 10	140^33^ ± 60	1	
ɣ-Aminobutyric acid (GABA)	(@ 2.91 ppm) k0 = 1030, kb = 5.68e + 10	17.2 ± 10.1	6900 ± 90	—	3	1.5^50,55^
Taurine (Tau)	(@ 3.18 ± 0.04 ppm) k0 = 10970, kb = 3.60e + 11	—	49600 ± 1470	—	3	1.0/2.1^53^ WM/GM
L-Glutamic acid (Glu)	(@ 3.20 ppm) k0 = 2790, kb = 4.50e + 10	6.9 ± 0.7	7480 ± 90	5500^2^ ± 500	3	6.6/11.5^50,54^ WM/GM
L-glutamine (Gln)	1 (@ 2.15 ppm) k0 = 12, kb = 6.44e + 07	13.8 ± 1.1	17 ± 2	—	1	3^17^
	2 (@ 2.87 ppm) k0 = 7, kb = 3.81e + 08		49 ± 2	—	1	
	3 (@ 3.18 ppm) k0 = 7880, kb = 1.31e + 11		22880 ± 530	—	3	
N-acetyl-L-aspartate (NAA)	(@ 3.33 ppm) k0 and kb are undetermined	—	~0	—	1	10.1/12.0^50,52^ WM/GM
Mobile proteins and peptides (mAmides)	(@ 3.50 ppm) k0 = 0, kb = 2.22e + 08 k0 and kb were obtained from pH dependence of the exchange rate derived in^20^.	—	—	22^20^	—	72^20^

The resonance frequencies of the labile protons are relative to the water resonance. Unless shown otherwise, the error in the fitted resonance frequencies was less than 0.01 ppm (95% confidence interval). The errors in the measured exchange rates and T2′s of labile protons represent 95% confidence intervals.

Presented below are the results for all metabolites grouped together depending on the type of exchanging protons.

### Hydroxyl protons

The hydroxyl protons of both glucose (Glc) and myo-inositol (MI) have their resonance frequencies close to water (Δω up to 1.40 ppm downfield from the bulk water resonance) with an exception for the α- and β-protons of Glc (at position **1**, see Table 1), the resonance frequencies of which are 2.18 ppm and 2.88 ppm, respectively. The composition of D-glucose at 37 °C found by fitting full BME is in line with literature^19^, i.e. 42.7% and 57.3%, for the α- and β-isomer, respectively. Interestingly, the hydroxyl proton at position **1** of the Glc β-isomer (Δω = 2.88 ppm, *k* = 4750 Hz) has a faster exchange rate than that of the Glc α-isomer (Δω = 2.18 ppm, *k* = 3860 Hz). All six hydroxyl protons of MI have an average resonance frequency of 1.00 ppm. The exchange rates of the hydroxyl protons of Glc are higher compared to those of MI, except for the Glc hydroxyl proton at position **5** resonating at 0.74 ppm.

### Guanidinium protons

The four magnetically equivalent guanidinium protons of creatine (Cr) resonate at 2.00 ppm and have an exchange rate of 810 Hz, whereas the resonance frequencies of the guanidinium protons of phosphocreatine are 1.93 ppm (position **1**, *k* = 67 Hz) and 2.64 (position **2**, *k* = 126 Hz).

### Amino protons

The amino protons of ɣ-aminobutyric acid (GABA), taurine (Tau), glutamate (Glu) and glutamine (Gln) have their resonance frequencies around 3.00 ppm and are characterized with fast exchange rates, i.e., *k* ≈ 7 kHz for GABA and Glu, and *k* ≫ 10 kHz for Tau and Gln.

### Amide protons

The amide protons of Gln resonate at 2.15 ppm and 2.87 ppm and are characterized with a slow exchange, *k* = 17 Hz and *k* = 49 Hz, respectively. The single amide proton of N-acetyl-L-aspartate (NAA) resonating at ca. 3.33 ppm was measured to have a very slow exchange rate *k ≈* 0 Hz. Based on the literature, the amide protons originating from mobile proteins and peptides (mAmides) resonate at 3.5 ppm and have an exchange rate *k* = 22 Hz^20^.

### BME simulations

Figure 1 shows the results of transmit field amplitude (B1) and saturation time optimization using BME at the field strength of 7T (see Figs 20S–25S in Supplementary Material for other fields strengths) for MI, Cr, PCr, Glu and mAmides at their corresponding resonance frequencies. To reach the maximum CEST effect size, the slow exchanging protons such as guanidinium proton (Δω = 2.64 ppm) of PCr and amide protons of mAmides require a low B1 (<1.5 µT) and a long saturation time (>2.5s). For the intermediate exchanging protons, e.g. hydroxyl protons of MI and guanidinium protons of Cr, a higher B1 (ca. 2 µT) and a shorter saturation (<2 s) are needed. The fast exchanging amine protons of Glu require a high B1 (ca. 6 µT) and a short saturation time (<1s). The saturation schemes yielding the largest CEST effects for the field strengths of 3T, 4.7T, 7T, 9.4T, 11.7T, and 14.1T are summarized in Table 2.

**Table 2:** The CEST-prepulse parameters yielding the largest CEST effects (pH = 7) for MI, Cr, PCr, Glu and mAmides at the field strengths of 3T, 4.7T, 7T, 9.4T, 11.7T and 14.1T, based on the measured metabolic parameters (see Table 1).

Metabolites	B1 [µT]						Saturation time [s]					
	3T	4.7T	7T	9.4T	11.7T	14.1T	3T	4.7T	7T	9.4T	11.7T	14.1T
MI (1.00 ppm)	1.2	1.7	2.1	2.3	2.4	2.3	0.5	0.5	0.5	0.6	0.7	0.8
Cr (2.00 ppm)	1.7	2.0	2.1	2.2	2.3	2.3	0.9	1.3	1.8	2.4	2.6	2.8
PCr (2.64 ppm)	1.0	1.2	1.3	1.4	1.4	1.4	4.6	5.0	5.0	5.0	5.0	5.0
Glu (3.20 ppm)	4.3	5.5	6.8	7.5	8.2	7.9	0.5	0.5	0.5	0.6	0.6	0.7
mAmides (3.50 ppm)	0.6	0.7	0.8	0.8	0.9	0.9	5.0	5.0	5.0	5.0	5.0	5.0

Figure 2 shows normalized metabolic contributions to the brain Z-spectra in grey matter (GM) as a function of pH at the resonance frequencies of MI, Cr, PCr, Glu and mAmides. The overall trend is that an increase in pH triggers an increase in CEST contributions from PCr and mAmides and a decrease in CEST contributions from other metabolites except Cr, the contribution of which is maximum at pH = 7. With the corresponding saturation schemes yielding the largest CEST effects (see Table 2), the maximum contributions to the GM Z-spectra at 7 T and pH = 7 for MI (at 1.00 ppm), Cr (at 2.00 ppm), PCr (at 2.64 ppm), Glu (at 3.20 ppm), and mAmides (at 3.50 ppm) are 19%, 38%, 21%, 59%, and 83%, respectively.

In Fig. 3, normalized metabolic contributions to the GM Z-spectra (pH = 7) are plotted as a function of frequency offset. Metabolites with slower exchanging protons (Cr, PCr and mAmides) tend to have more sharp frequency profiles. A careful analysis of the metabolic contributions as a function of pH (Fig. 2) and frequency offset (Fig. 3) reveals that regardless of both parameters, five main contributors, i.e. MI, Cr, PCr, Glu and mAmides, account for ca. 90% of CEST contrast in brain Z-spectra.

Figure 4 shows the comparison of CEST effects (pH = 7) from the individual labile protons of the major brain metabolites (see Table 1) at the resonance frequencies of MI, Cr, PCr, Glu and mAmides at the field strengths of 3T, 4.7T, 7T, 9.4T, 11.7T and 14.1T. CEST effects of all labile protons increase with the static magnetic field and regardless of the field strength, MI, Cr, PCr, Glu and mAmides account for ca. 90% of CEST contrast in brain Z-spectra. CEST effect size of fast exchanging protons has much stronger field strength dependence when compared to slow exchanging protons. The changes in CEST effect size (with the saturation schemes yielding the largest CEST effects, see Table 2) from 3T to 14.1T for MI, Cr, PCr, Glu, and mAmides at their corresponding resonance frequencies are 79%, 74%, 56%, 79%, and 40%, respectively. See Figs 26S–27S in Supplementary Material for Glc CEST effects.

## Discussion

The aim of this work was to identify the major brain metabolites that contribute to downfield CEST phenomena in brain Z-spectra at different field strengths and pH. To address this, we quantified exchange rate constants of major brain metabolites and used the signatures of these metabolites in BME at different field strengths and pH. We show that, regardless of field strengths and pH levels, five metabolites, i.e. myo-inositol, creatine, phosphocreatine, glutamate and mobile amides, account for ca. 90% of CEST contras in Z-spectra downfield from the bulk water resonance.

The exchange rate constant (*k*) is arguably the single most important parameter for optimizing detection of metabolites in CEST experiments. This parameter governs the labeling efficiency of labile protons^10^ and determines the optimum parameter settings for the CEST-prepulse^21^ for *in-vivo* imaging of the metabolites. Multiple BME simplified approaches have been suggested for exchange rate measurements of labile protons^22–24^. However, these simplified methods are not valid for fast exchanging protons^24,25^. Previously, resonance frequencies and exchange rates (line broadening in ^1^H-NMR) for many metabolites with a single exchanging site were determined at 11.7T at 25 °C^26^. Others studied metabolic variations with CEST (magnetization transfer ratio analysis) in human breast cancer cell lines at 11.7T at 37 °C^27^. In a recent review, simulations for four downfield CEST effects, i.e. for myo-inositol, creatine, glutamate and amides, were performed at the field strengths of 3T, 7T and 11.7T^28^. However, comprehensive measurements of metabolic exchange rates at 37 °C and an extensive analysis of pH-dependent metabolic contributions to Z-spectra in the brain for multiple field strengths are not yet available in the literature.

Therefore, in this work, we chose to quantify exchange rates by fitting full BME to metabolic Z-spectra obtained at an ultra-high field strength of 14.1T at an *in-vivo* relevant temperature of 37 °C in the physiological pH range 6.4–7.3. The high field strength yields a high chemical shift dispersion, which allows for the precise measurements of the field strength-independent parameters such as resonance frequency (with respect to water) and exchange rate. To increase precision and reduce parameter correlation, which is especially important for the metabolites with multiple exchanging sites, BME were fitted simultaneously to metabolic Z-spectra (for all pH levels) obtained at multiple B1 saturation levels of the CEST prepulse. Once metabolic CEST parameters are known, BME can be used to yield metabolic CEST signatures at any field strength and at any pH. In this work, we used metabolic CEST signatures to study metabolic contributions to brain Z-spectra over a variety of main field strengths, i.e. 3T, 4.7T, 7 T, 9.4T, 11.7T, and 14.1T, in the physiological pH range.

Out of the five hydroxyl proton resonances identified for Glc^29^, three resonances from four protons (from 0.7 ppm to 1.4 ppm) are suitable for *in vivo* imaging of Glc due to favorable exchange rates. The relatively low *in vivo* concentration of Glc, however, necessitates its exogenous administration for a detectable CEST effect, which has been exploited for imaging of the blood brain barrier disruption in brain tumors^30,31^.

MI is one of the osmolytes in the central nervous system^32^ and MI mapping may help in diagnosis and treatment monitoring of many neuropsychiatric conditions. In the first publication on MI CEST in human brain at 7T^7^, a resonance frequency of 0.6 ppm and exchange rate of 600 Hz (pH = 7.4) were reported for MI hydroxyl protons. These results are different from those of our study (Δω = 1.00 ppm and *k* = 2090 Hz), which we attribute to the fact that authors used a simplified equation for exchange rate measurement and an asymmetry analysis (MTRasym) for determining the resonance frequency, both of which are not accurate for fast exchanging protons. The peak CEST effect in MTRasym for fast exchanging protons (*k* > Δω) appears to be shifted closer to the bulk water resonance, which can be mistaken for its real resonance frequency. Glu is a nice example of this effect, with the resonance of its amino protons being at Δω = 3.20 ppm, whereas the peak CEST effect in MTRasym at 7T (pH = 7) appears at ca. Δω = 1.20 ppm^2^. The resonance frequency of MI hydroxyl protons (Δω = 1.00 ppm) lies in the frequency range used for Glc imaging. However, the endogenous concentration of MI is much higher than for Glc. This, combined with six protons in MI resonating at the same frequency, makes MI a stronger contributor to CEST contrast at 1.00 ppm than Glc^7^. Rather than quantifying the MI CEST effect at a single frequency, an increase in sensitivity can be achieved if an area from ca. 0.2–1.1 ppm is used.

The results of our measurements of resonance frequency and exchange rate for the guanidinium protons of Cr and PCr are close to the literature values^33^. Cr is converted by the enzyme creatine kinase (CK) to PCr, which acts as a temporal energy buffer in brain and muscles, according to the reaction^34^:

$$ATP+creatine\frac{CK}{ < ======= > }PCr+ADP+{H}^{+}$$

Our results suggest that the guanidinium proton of PCr should also give a detectable CEST effect further downfield from water (Δω = 2.64 ppm). An assumption of a Cr/PCr ratio of 1 in the brain^17^ yields the following ratios of their CEST effects (Cr/PCr at pH = 7) at Δω = 2.00 ppm (with the saturation scheme yielding the largest CEST effect for Cr): 2.5 (3T), 3.2 (4.7T), 3.3 (7T), 3.5 (9.4T), 3.8 (11.7T) and 3.8 (14.1T); and at Δω = 2.64 ppm (with the saturation scheme yielding the largest CEST effect for PCr): 1.1 (3T), 1.4 (4.7T), 1.6 (7T), 1.8 (9.4T), 1.9 (11.7T) and 1.9 (14.1T). In skeletal muscle, where at least 70% of total Cr exists in its phosphorylated form, i.e. PCr^35,36^, the ratios of their CEST effects (Cr/PCr at pH = 7) at Δω = 2.00 ppm (with the saturation scheme yielding the largest CEST effect for Cr) become: 1.1 (3T), 1.4 (4.7T), 1.4 (7T), 1.5 (9.4T), 1.6 (11.7T) and 1.6 (14.1T); and at Δω = 2.64 ppm (with the saturation scheme yielding the largest CEST effect for PCr): 0.5 (3T), 0.6 (4.7T), 0.7 (7T), 0.8 (9.4T), 0.8 (11.7T) and 0.8 (14.1T). Total Cr has recently been measured experimentally in mouse brain with ^1^H-MRS at 11.7T and the CEST effect at 2.00 ppm at 11.7T (2 µT B1 amplitude and 2 s saturation) was estimated to consist of ca. 20% protein and 80% total creatine, of which 83% Cr and 17% PCr^37^. From our estimates, CEST effect in the brain at 2.00 ppm at 11.7T (2.3 µT B1 amplitude and 2.6s saturation) consists of 79% Cr and 21% PCr, which is in line with the recent study^37^. Cr and PCr constitute a coupled cellular energy transport system^38^ and the possibility of simultaneous and independent detection of Cr and PCr by CEST could open up a new way of studying cellular energy transport system *in vivo*, which is currently limited to *in vitro* experiments in mitochondrial extracts, *in vivo* ^1^H/^31^P MRS, or using ^13^C enriched labeling^39^.

Glu is the major excitatory neurotransmitter in the brain, the abnormal variation of which is associated with many diseases of the central nervous system^40^. A method for *in vivo* Glu mapping at 7T was published before and a 75% Glu-weighted CEST-MRI contrast was reported. However, in the present study, the exchange rates of major brain metabolites were measured by fitting full BME to their Z-spectra, and the purity of Glu CEST contrast was found to be only 57%.

The findings of this study can be exploited in the design of novel CEST sequences and advanced Z-spectra processing algorithms, which is the initial step towards quantitative metabolic CEST imaging. Even though, the focus of this work was the brain tissue, the results can be reanalyzed for other tissues as well.

## Limitations

Low specificity due to overlapping CEST effects from multiple metabolites is one of the challenges towards quantitative CEST. In this work, we found CEST saturation schemes yielding the maximum CEST effects for many brain metabolites. However, it is important to realize that even these saturation schemes never generate a pure contrast for any particular metabolite. Therefore, we analyzed metabolic contributions to Z-spectra as a function of frequency offset.

Neither magnetization transfer (MT) nor upfield nuclear Overhauser enhancement (NOE) phenomena were included in the BME model, as there are multiple approaches to remove those effects from the Z-spectra^10,12,13^.

In this study, we analyzed downfield CEST effects from low molecular weight metabolites with an exception for the CEST effect at 3.5 ppm originating from the amide protons of mobile proteins and peptides. However, mobile proteins have other types of exchangeable protons, although in a lower concentration compared to the abundant amide protons^26,37,41^. As such, mobile proteins *in vivo* may have other downfield CEST effects in the range 0–4 ppm. For instance, the CEST effect at 2 ppm in mouse brain at 11.7T (2 µT B1 amplitude and 2 s saturation) was estimated to consist of ca. 20% protein and 80% total creatine^37^. To make matters worse, protein CEST effects *in vivo* may be dependent on protein conformational changes^42^, which is difficult to take into account in simulations. Therefore, all metabolic ratios presented in this work should be considered with the above information in mind.

The exchange rate constant of labile protons depends, among other factors, on their chemical environment, which can be highly catalytic^43^. Ideally, the composition of *in vitro* metabolic solutions should reflect the relevant *in vivo* chemical environment. Unfortunately, that is difficult to achieve as the precise cellular composition is unknown and is highly variable. Free phosphate and its derivatives with similar acid dissociation constant, i.e. adenosine triphosphate and phosphate monoesters, which are known to be predominant exchange catalysts in intracellular fluid^43^, amount to a concentration of 8.5 mM in brain and 13.0 mM in muscle tissue^43^. Therefore, in this work, we chose to use a 10 mM phosphate-buffered saline (PBS) as a medium for metabolic solutions.

## Methods

All metabolites were purchased from Sigma-Aldrich and used as received without further purification.

### Determination of resonance frequency, exchange rate and T2 of labile protons

The exchange rate measurements for the labile protons of the major brain metabolites, i.e. glucose (Glc), myo-inositol (MI), creatine (Cr), phosphocreatine (PCr), gamma-aminobutyric acid (GABA), glutamine (Gln), N-acetyl-aspartate (NAA), taurine (Tau), and glutamate (Glu), were performed in 25 mM or 50 mM phantoms (pH: 6.4, 6.7, 7.0, and 7.3, 10 mM PBS as a medium) at 37 °C on a 600 MHz NMR-spectrometer (Bruker). Coaxial inserts (Wilmad^®^) were used for measurements: D2O and sample were put in outer and inner tube, respectively. Metabolic Z-spectra were acquired with a 5 s rectangular pulse for magnetization preparation with a post-readout T1 recovery of 25 s. Water T1 relaxation times (T1water) in phantoms were measured by inversion recovery (IR) at the following inversion times (IT): 4 ms, 30 ms, 100 ms, 200 ms, 500 ms, 1 s, 2 s, 4 s, 8 s, 12 s, 16 s, 20 s, and 25 s. For both CEST and T1 measurements, a flip angle of 10° was used. B1 saturation levels of the prepulse were optimized for a particular metabolite. To decouple correlated parameters, which is especially important for the metabolites with multiple exchanging sites, multi-pool BME were fitted to metabolic Z-spectra (for all pH levels), obtained at multiple B1 levels, simultaneously (see Figs 1S–9S in Supplementary Material). Multiple B1 levels for the CEST prepulse yield a varying level of labeling efficiency for exchangeable protons, which in turn increases precision of the BME fitting algorithm. The fixed parameters in BME were proton concentration (a product of metabolic concentration and number of exchangeable protons) and water T1 (measured by IR), whereas lower and upper bounds were set on the other parameters, i.e. proton resonance frequency (with respect to the water resonance), exchange rate and T2 relaxation of labile protons. The initial guesses for the resonance frequencies were based on the corresponding metabolic ^1^H-NMR spectra (see Figs S10–S18 in Supplementary Material). To reduce the number of degrees of freedom, the resonance frequency and T2 of labile protons for a particular metabolite were assumed to be the same across all pH values. Also, for the metabolites with multiple exchanging sites, all labile protons were assumed to have the same T2 relaxation times. To produce a global solution, fitting was performed with a MultiStart (with 100 random initial guesses) algorithm with lsqcurvefit solver in MATLAB. The global solution was used as an initial guess with lsqnonlin solver in MATLAB to produce a jacobian matrix, which was subsequently used to generate 95% confidence intervals on the fitted parameters.

### Exchange rate constants measurements

The exchange rate between exchangeable protons and water is defined as follows^43^:

1 $$k={k}_{0}+{k}_{a}[H3{O}^{+}]+{k}_{b}[H{O}^{-}]+{\sum }^{}{k}_{c}{(catalyst)}^{n}$$

where *k*_0_ is the exchange rate constant due to spontaneous catalysis; *k*_*a*_, *k*_*b*_ and *k*_*c*_ are the exchange rate constants due to catalysis by acid, base, and other exchange catalysts, respectively.

The exchange rate measurements were done in metabolic solutions with a pH ≥ 6.4, therefore we assumed that *k*_*b*_ ≫ *k*_*a*_, and hence *k*_*a*_ = 0. We did not fit separately for the catalytic term (*k*_*c*_), and so *k*_0_ may contain a contribution from the *k*_*c*_ term. The rate constants *k*_0_ and *k*_*b*_ for the exchangeable protons of each metabolite were obtained by fitting Equation 1 (*k*_*a*_ = 0, *k*_*c*_ = 0) to the experimentally determined plot of the exchange rate (*k*, obtained from BME above) versus pH.

### Optimization of CEST-prepulse parameters

The optimization of CEST-prepulse parameters, i.e. B1 amplitude of a rectangular pulse and saturation time (based on 25 ms pulses and 100% duty cycle), for imaging of MI, Cr, PCr, Glu and mAmides was done in BME at the field strengths of 3T, 4.7T, 7T, 9.4T, 11.7T, and 14.1T. Multi-pool (bulk water protons and the labile protons of all metabolites) BME were solved numerically^44^ using the following bulk water relaxation times^45–49^: T1/T2 = 0.9 s/70 ms (at 3T), T1/T2 = 1.07 s/60 ms (at 4.7T), T1/T2 = 1.2 s/40 ms (at 7T), T1/T2 = 1.66 s/37 ms (at 9.4T), and T1/T2 = 1.74 s/27 ms (at 11.7T) for white matter (WM); T1/T2 = 1.45 s/90 ms (at 3T), T1/T2 = 1.47 s/73 ms (at 4.7T), T1/T2 = 1.8 s/55 ms (at 7T), T1/T2 = 2.1 s/42 ms (at 9.4T), and T1/T2 = 2.11 s/37 ms (at 11.7T) for grey matter (GM). The water relaxation times at 14.1T were obtained by fitting and extrapolating the above literature values (see Fig. 19S in Supplementary Material): T1/T2 = 1.98 s/20.4 ms (WM) and T1/T2 = 2.45 s/25.9 ms (GM). See Table 1 for other parameters used in the simulations. T2 of labile protons of taurine could not be fitted reliably (large 95% confidence interval) and was assumed to be 10 ms. T2 of labile protons of mAmides was set to 10 ms as well.

The exchange rates used in BME were obtained by using the experimentally determined exchange rate constants and Equation 1. The B1 amplitude and the saturation time yielding the largest CEST effect are defined as follows:

2 $$CEST=S(\Delta \omega ,\,Ma=0)-S(\Delta \omega ,\,Ma=1)$$

where *S(*Δω,*Ma)* is the simulated signal in the z-spectrum at the resonance frequency Δω, and *Ma* is the simulated amplitude of CEST effect.

### Generation of stacked bar plots

Normalized CEST contribution for a particular metabolite in stacked bar plots was calculated as follows:

3 $$CESTnor{m}_{i}=\frac{CES{T}_{i}}{{\sum }_{i=1}^{n}CES{T}_{i}}$$

where *CEST*_*i*_ is the CEST effect size for a particular metabolite at a specific frequency, is the sum of CEST effects for all metabolites at the same frequency.

## Supplementary information

Supplementary material

## Data Availability

The datasets generated during and/or analyzed during the current study are available from the corresponding author on reasonable request.
