# Supplementary material for: Analysis of chemical exchange saturation transfer contributions from brain metabolites to the Z-spectra at various field strengths and pH
Source: Sci Rep. 2019 Jan 31;9:1089. doi: 10.1038/s41598-018-37295-y (PMC6355971; doi:10.1038/s41598-018-37295-y)
Supplement: Supplementary file 1 — Supplementary material [file 41598_2018_37295_MOESM1_ESM.docx]

**Supplementary material**

**Analysis of chemical exchange saturation transfer contributions from brain metabolites to the Z-spectra at various field strengths and pH**

Vitaliy Khlebnikov, Wybe J.M. van der Kemp, Hans Hoogduin, Dennis W. J. Klomp and Jeanine J. Prompers

**BME fitting to metabolic CEST spectra obtained at 14.1T**

Below are examples of the Bloch-McConnell equations (BME) fits to the metabolic CEST spectra (pH=6.4, 6.7, 7.0 and 7.3) recorded on a 600 MHz NMR spectrometer at 37 °C. The fits to the exchange rates were obtained using the following optimization function:

$k=k0+kb\left[ {HO}^{-} \right]$ (S1)

where *k_0_* and *k_b_* are the exchange rate constants due to the spontaneous and base catalysis, respectively.

**Glucose (Glc 25 mM)**


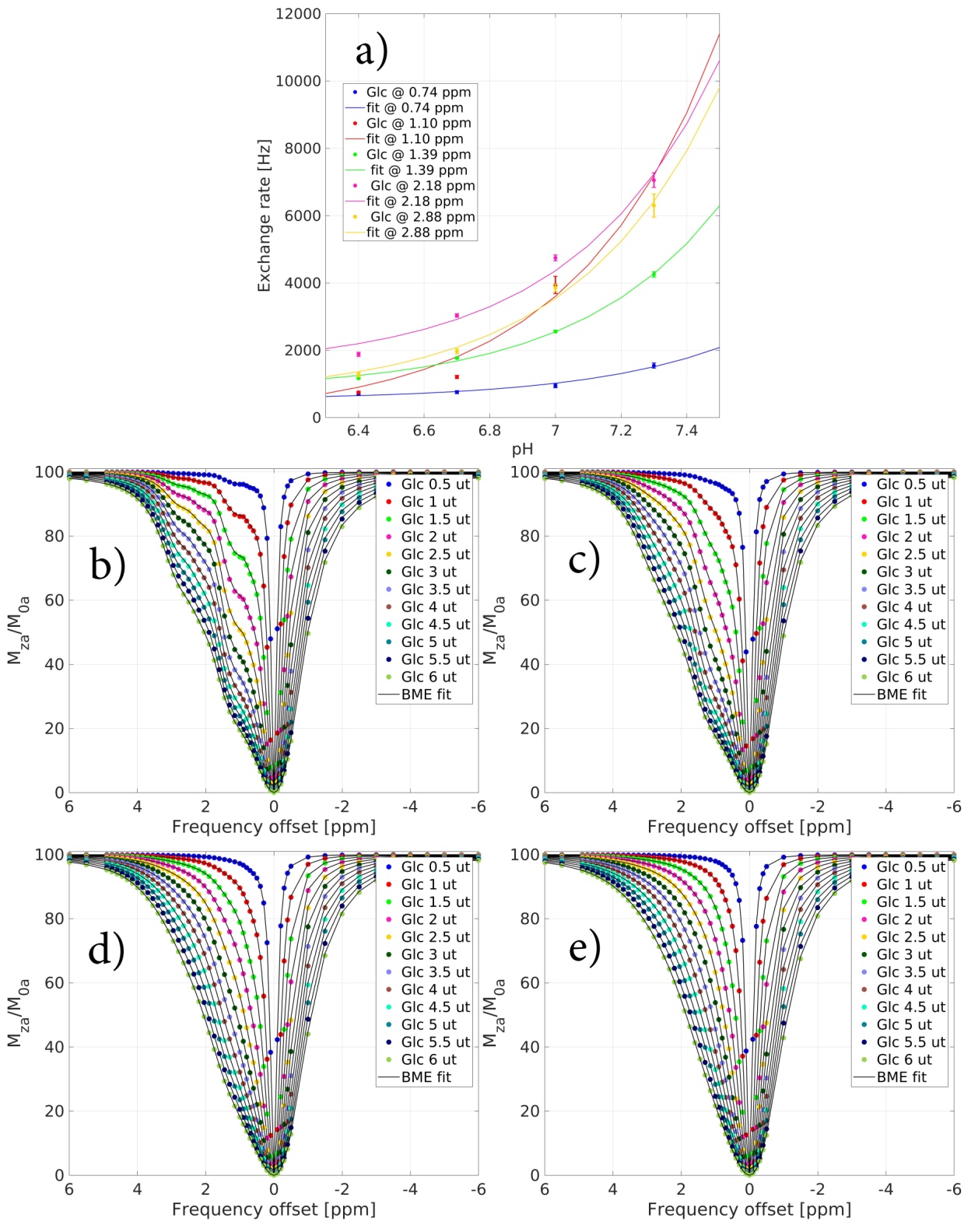


Fig. 1S. a) The experimentally measured exchange rates (95% confidence interval on error bars) of Glc exchangeable protons and the corresponding fit of eq. S1; and b-e) Glc CEST spectra at a pH of 6.4, 6.7, 7.0 and 7.3, respectively, obtained at various B1 levels (see legend) and the corresponding BME fits.

**Myo-Inositol (MI 25 mM)**


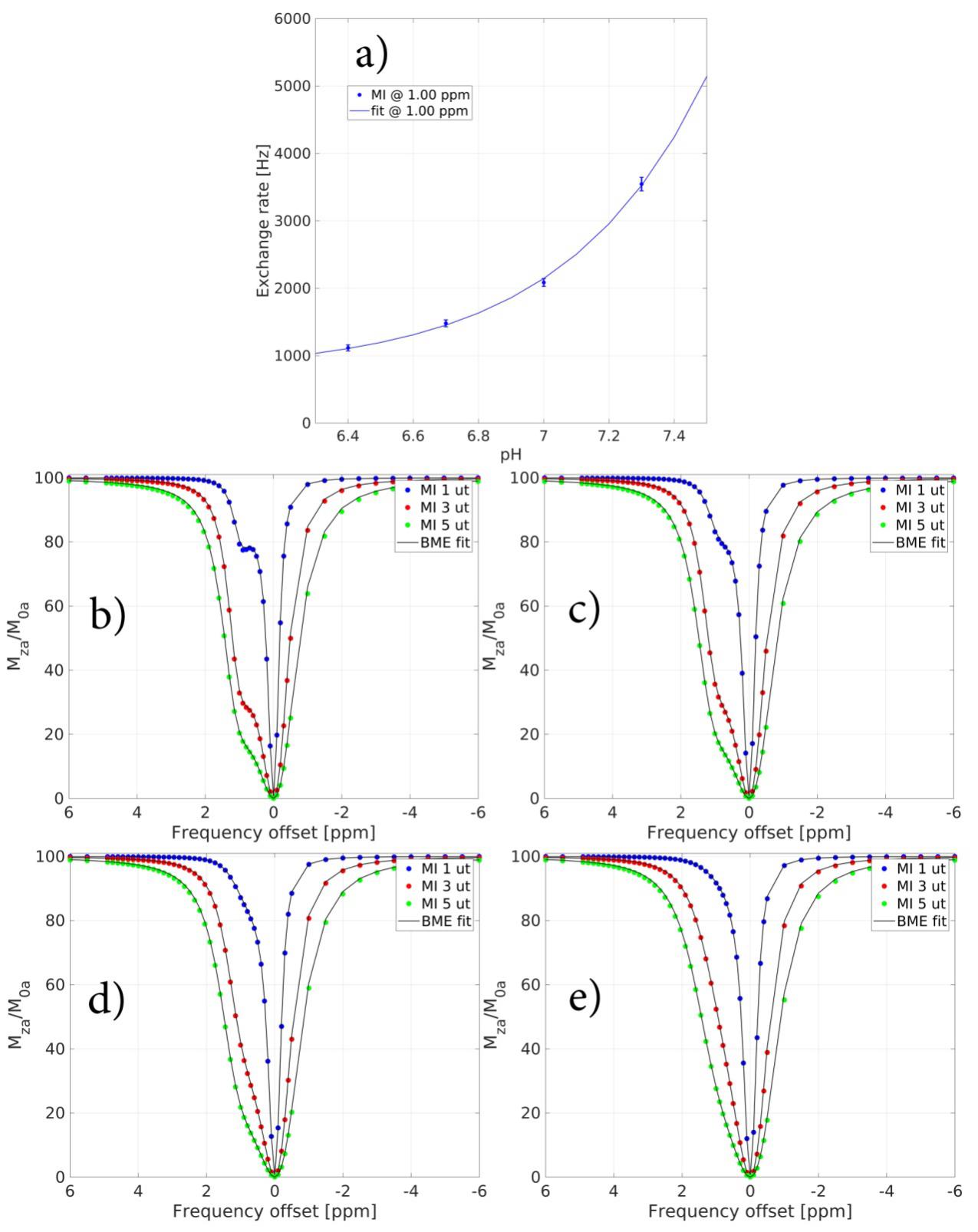


Fig. 2S. The experimentally measured exchange rates (95% confidence interval on error bars) of MI exchangeable protons and the corresponding fit of eq. S1; and b-e) MI CEST spectra at a pH of 6.4, 6.7, 7.0 and 7.3, respectively, obtained at various B1 levels (see legend) and the corresponding BME fits.

**Creatine (Cr 50 mM)**


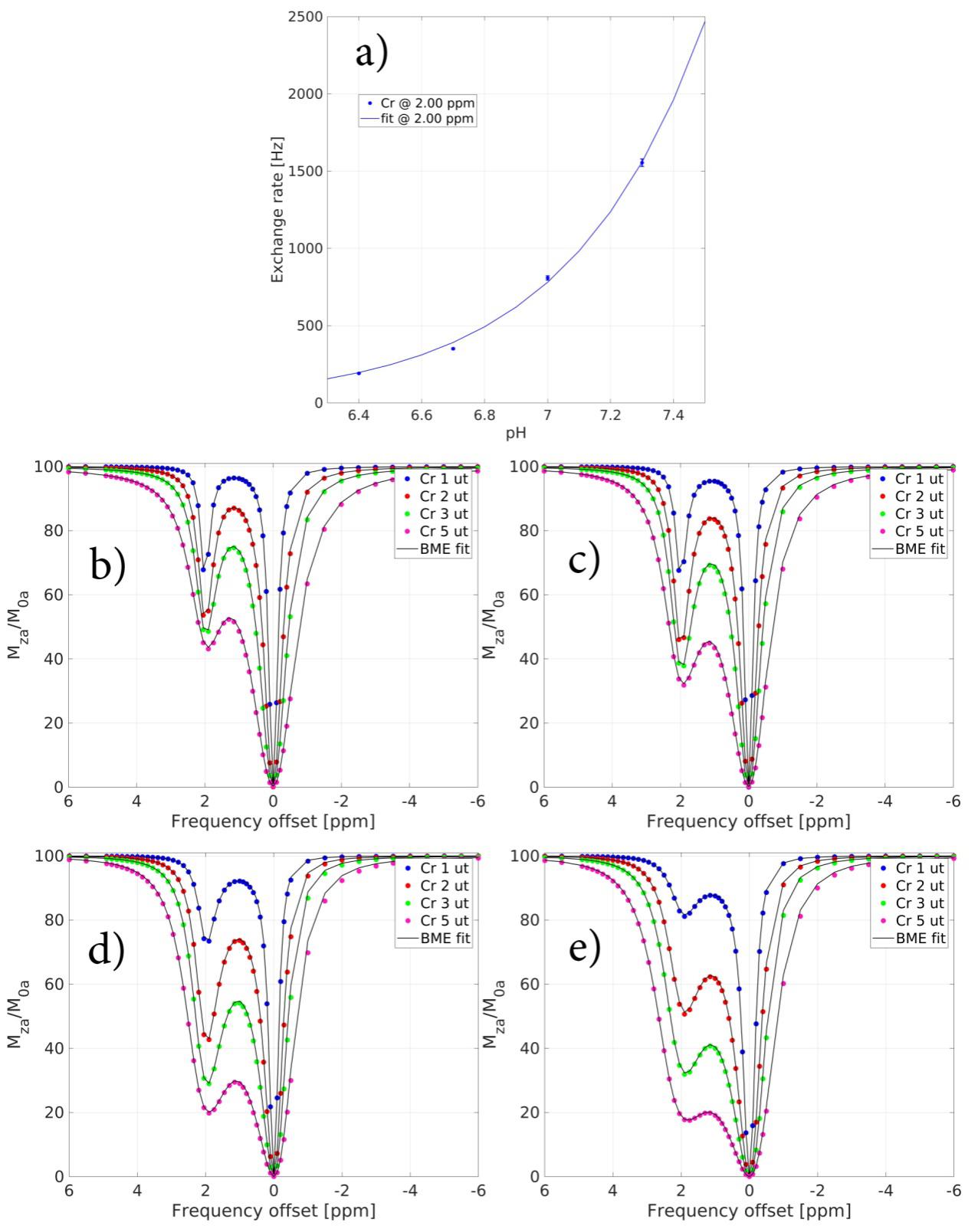


Fig 3S. a) The experimentally measured exchange rates (95% confidence interval on error bars) of Cr exchangeable protons and the corresponding fit of eq. S1; and b-e) Cr CEST spectra at a pH of 6.4, 6.7, 7.0 and 7.3, respectively, obtained at various B1 levels (see legend) and the corresponding BME fits.

**Phosphocreatine (PCr 50 mM)**


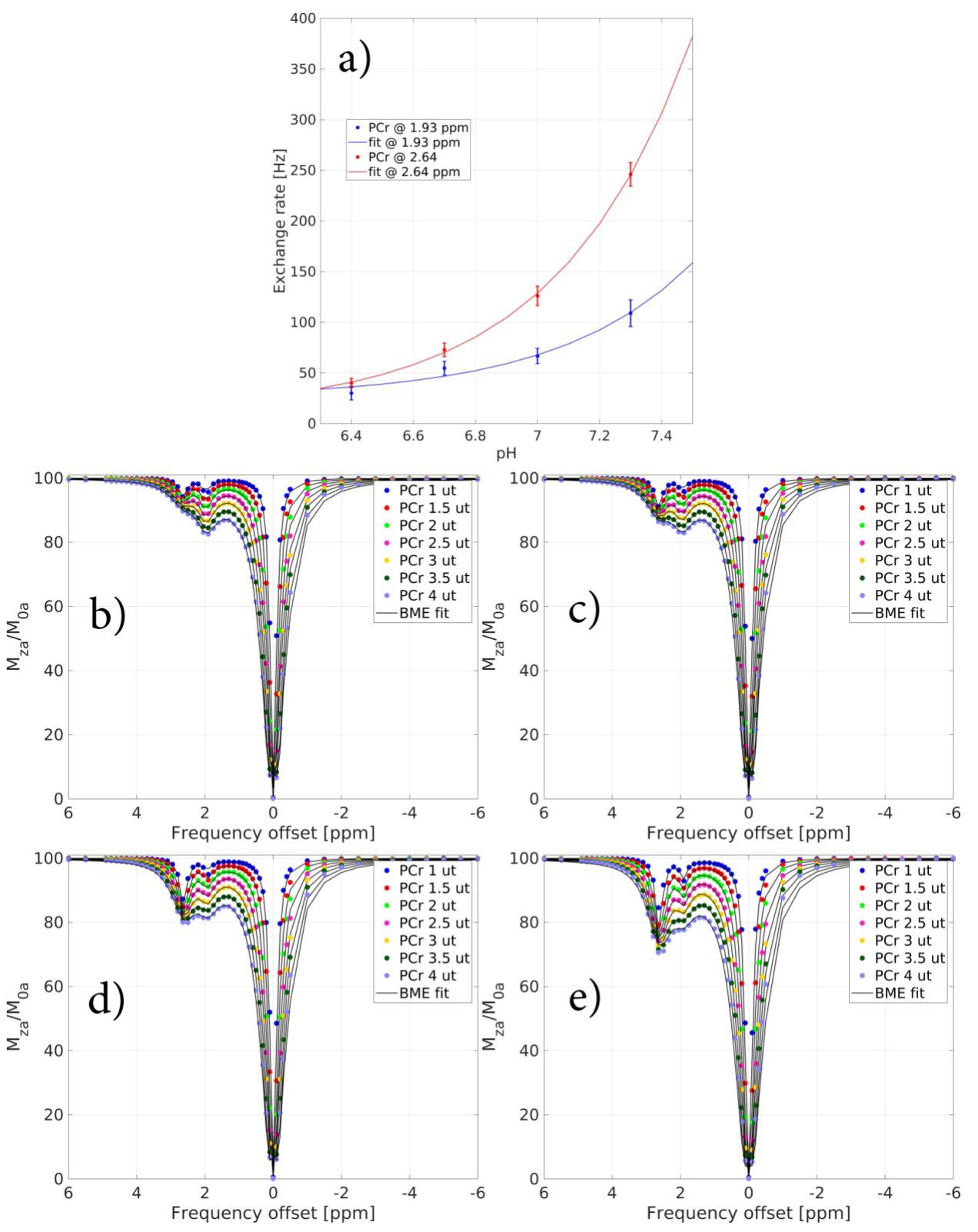


Fig 4S. a) The experimentally measured exchange rates (95% confidence interval on error bars) of PCr exchangeable protons and the corresponding fit of eq. S1; and b-e) PCr CEST spectra at a pH of 6.4, 6.7, 7.0 and 7.3, respectively, obtained at various B1 levels (see legend) and the corresponding BME fits.

**ɣ-aminobutyric acid (GABA 25 mM)**


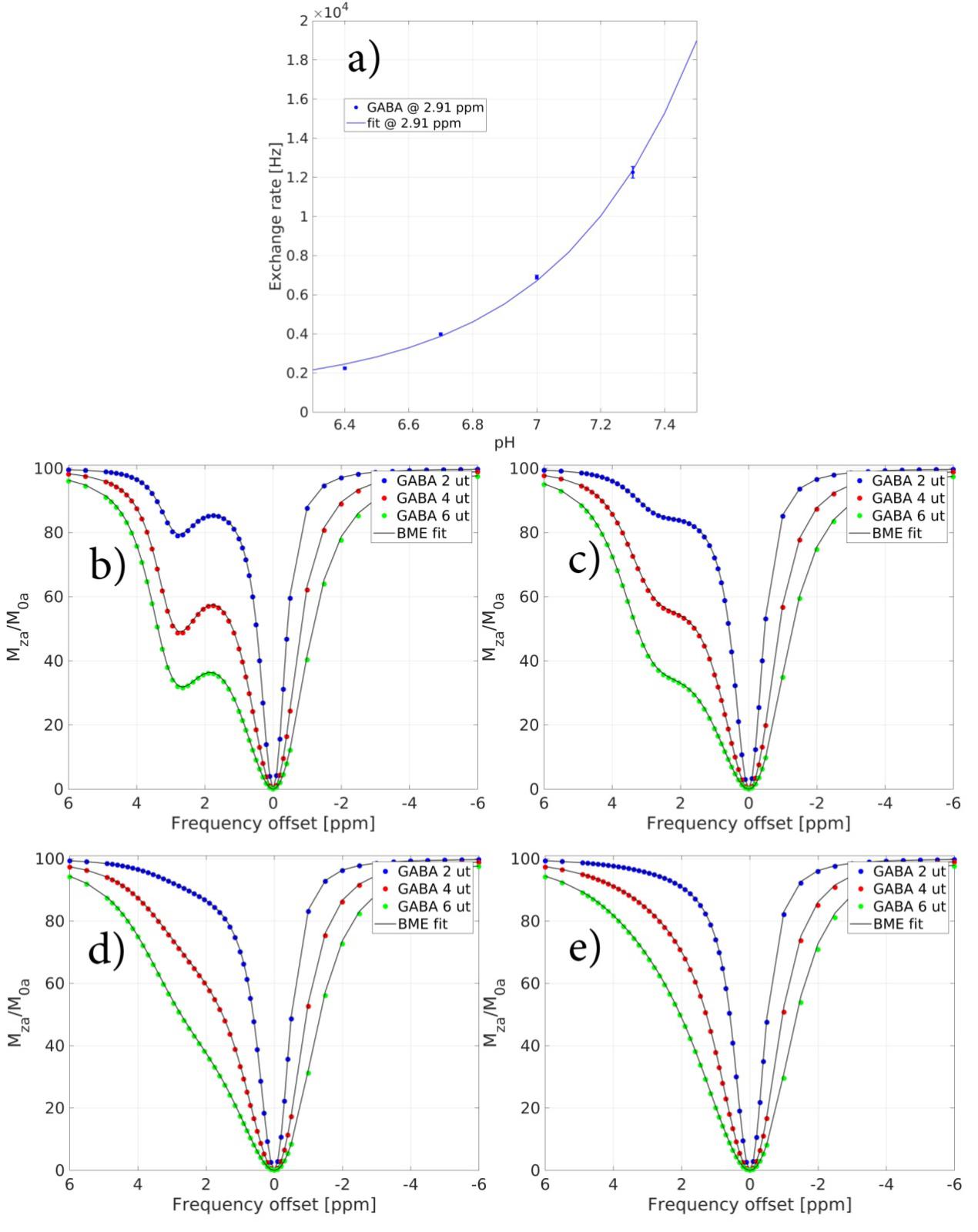


Fig 5S. a) The experimentally measured exchange rates (95% confidence interval on error bars) of GABA exchangeable protons and the corresponding fit of eq. S1; and b-e) GABA CEST spectra at a pH of 6.4, 6.7, 7.0 and 7.3, respectively, obtained at various B1 levels (see legend) and the corresponding BME fits.

**Taurine (Tau 25 mM)**


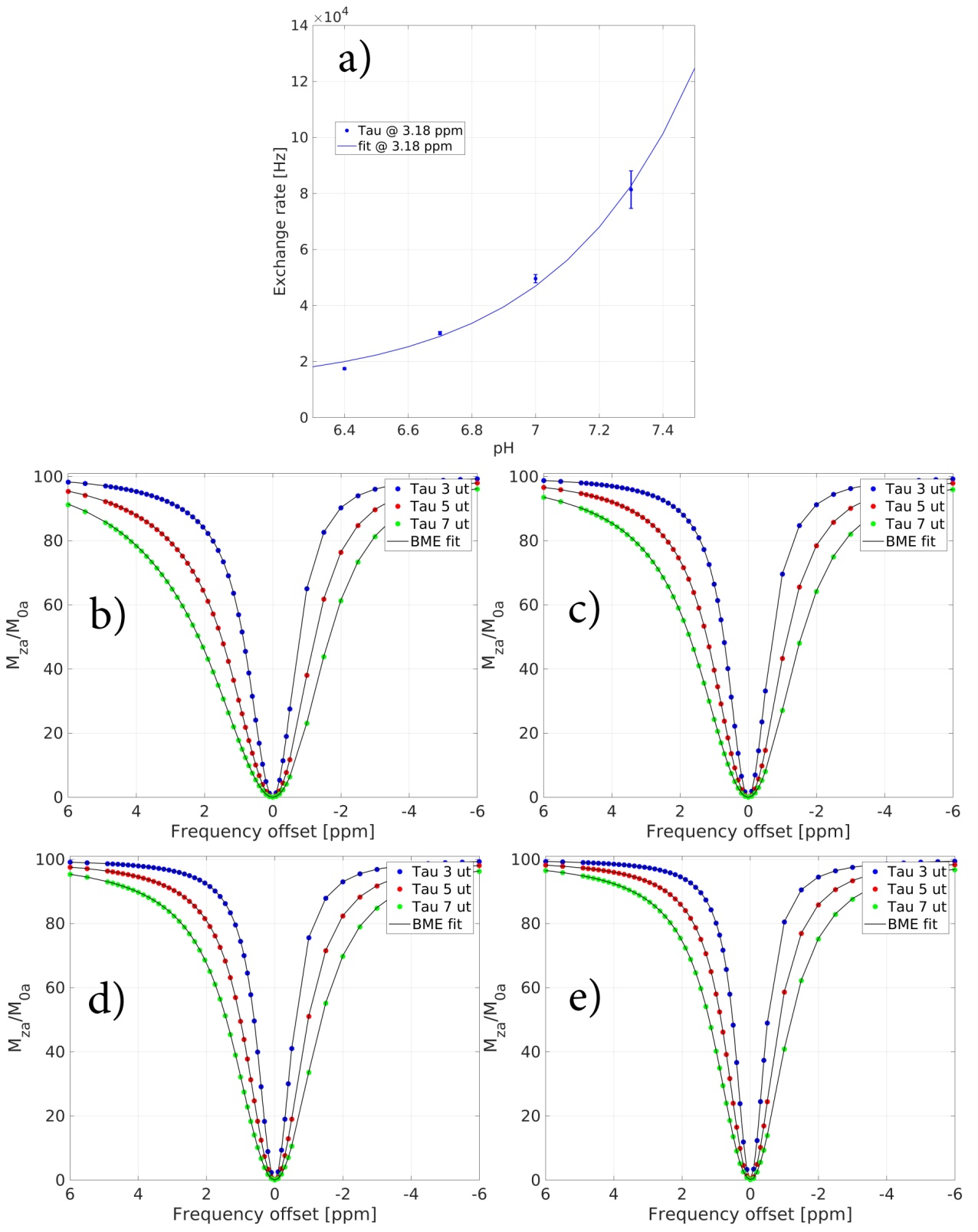


Fig 6S. a) The experimentally measured exchange rates (95% confidence interval on error bars) of Tau exchangeable protons and the corresponding fit of eq. S1; and b-e) Tau CEST spectra at a pH of 6.4, 6.7, 7.0 and 7.3, respectively, obtained at various B1 levels (see legend) and the corresponding BME fits.

**Glutamate (Glu 25 mM)**


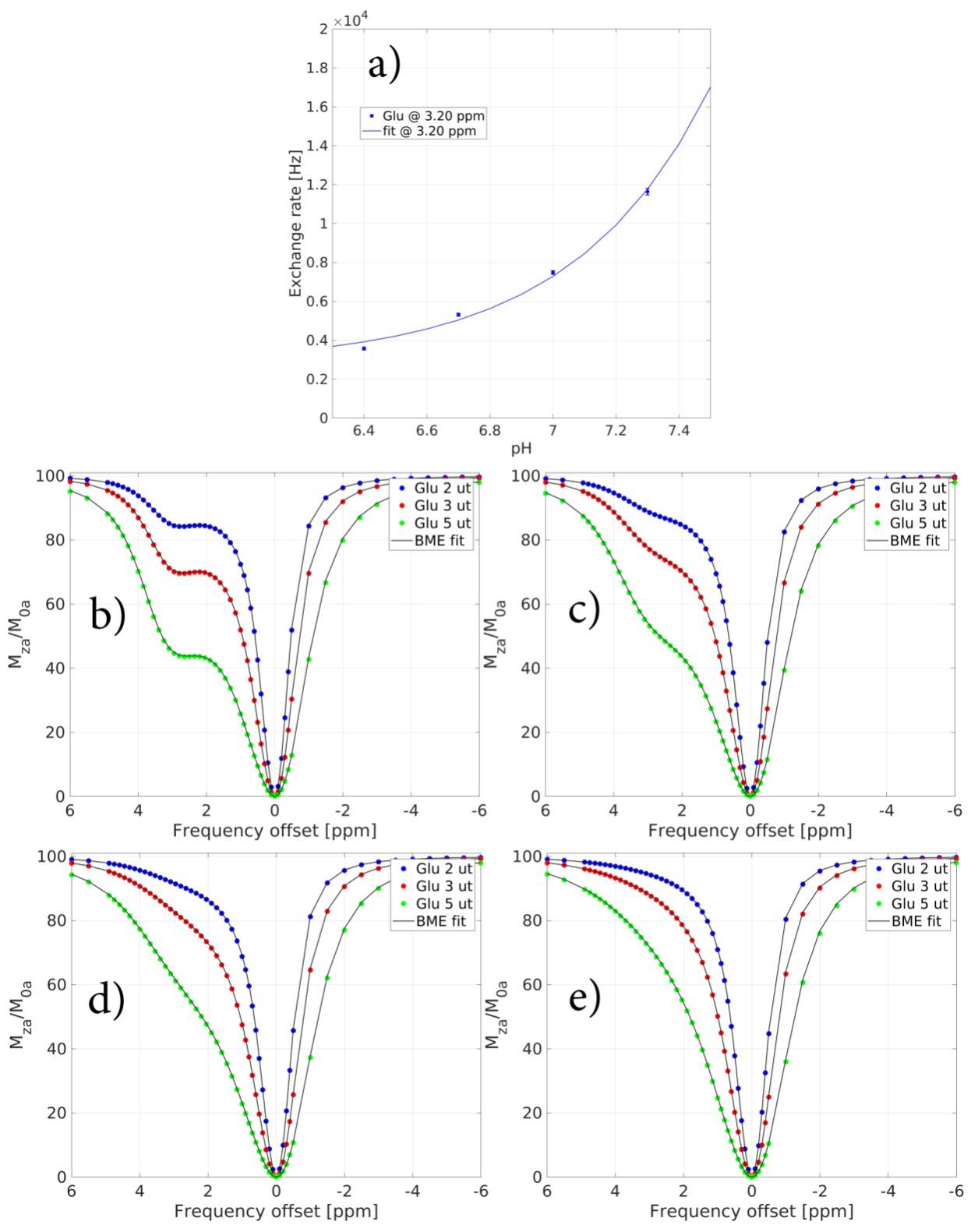


Fig 7S. a) The experimentally measured exchange rates (95% confidence interval on error bars) of Glu exchangeable protons and the corresponding fit of eq. S1; and b-e) Glu CEST spectra at a pH of 6.4, 6.7, 7.0 and 7.3, respectively, obtained at various B1 levels (see legend) and the corresponding BME fits.

**Glutamine (Gln 25 mM)**


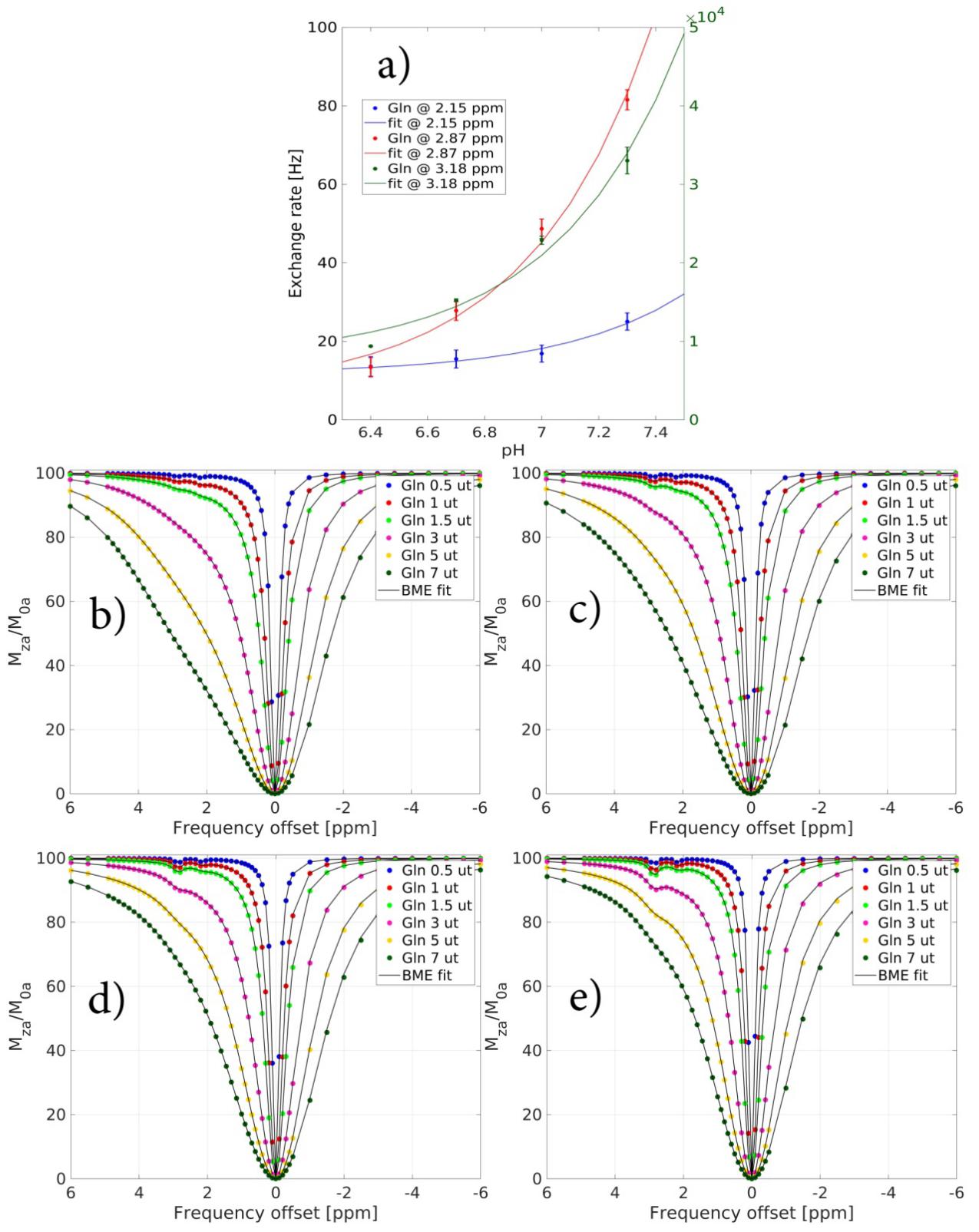


Fig 8S. a) The experimentally measured exchange rates (95% confidence interval on error bars) of Gln exchangeable protons and the corresponding fit of eq. S1; and b-e) Gln CEST spectra at a pH of 6.4, 6.7, 7.0 and 7.3, respectively, obtained at various B1 levels (see legend) and the corresponding BME fits.

**N-acetyl-aspartate (NAA 25 mM)**


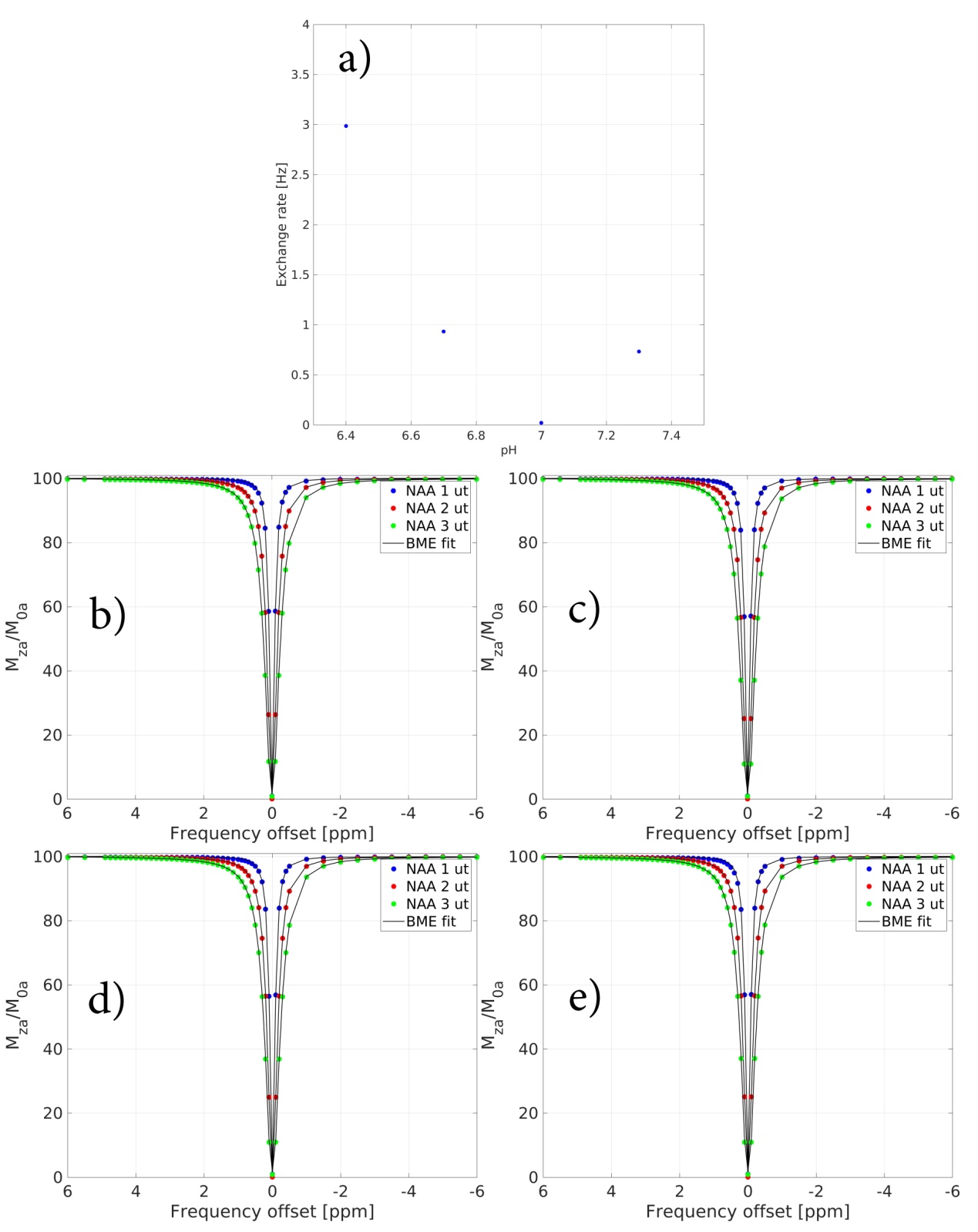


Fig 9S. a) The experimentally measured exchange rates of NAA exchangeable protons; and b-e) NAA CEST spectra at a pH of 6.4, 6.7, 7.0 and 7.3, respectively, obtained at various B1 levels (see legend) and the corresponding BME fits.

**600 MHz ^1^H-NMR spectra of metabolites**

Double distilled water was used for sample preparations. Before NMR acquisition, the metabolic solutions were filtered through a 200 µm membrane.

**Glucose (200 mM)**


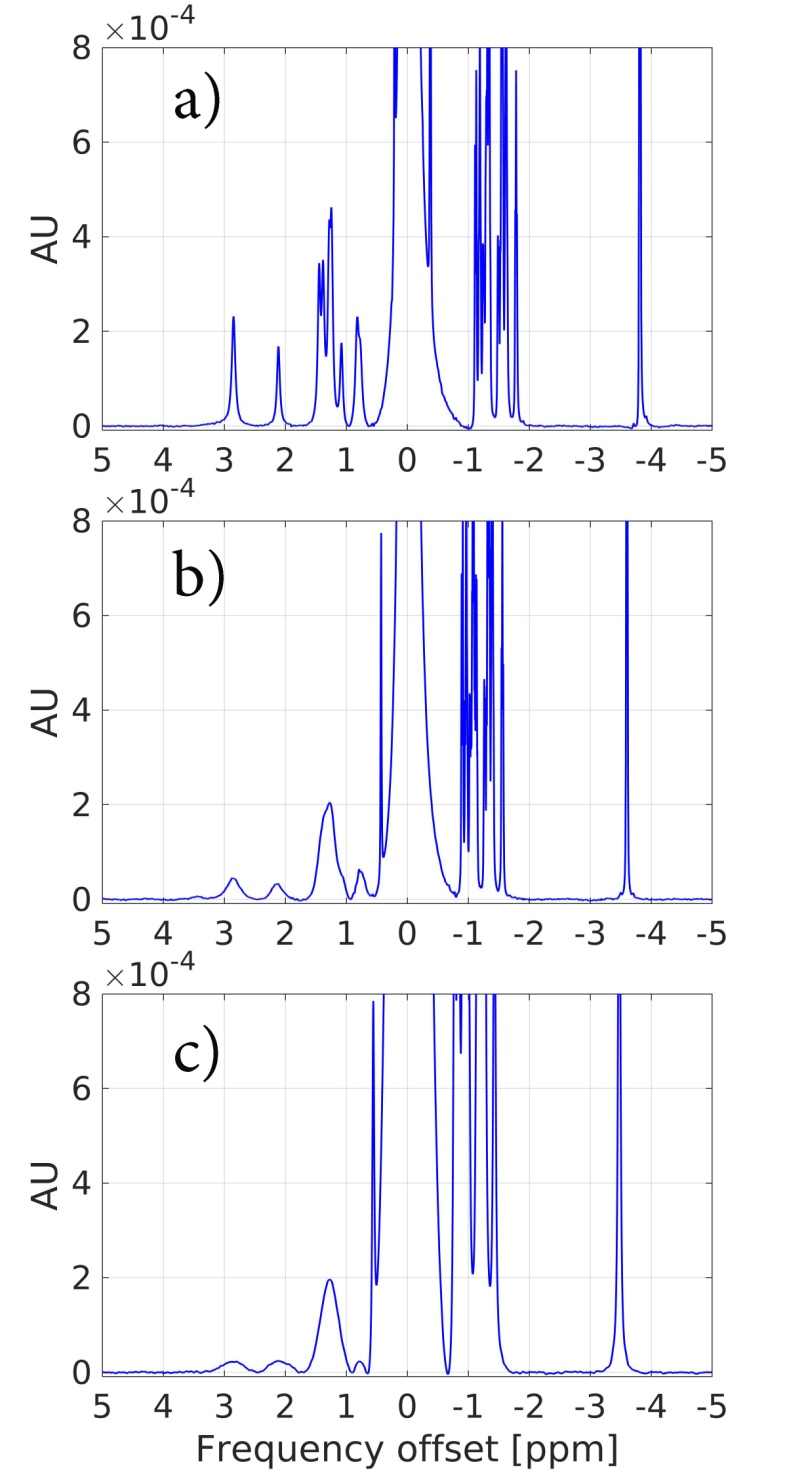


Fig. 10S. 1H-NMR spectrum of Glc water solution (200 mM, pH=6.5) at: a) 4 C°, b) 25 C° and c) 37 C°.

**Myo-Inositol (200 mM)**


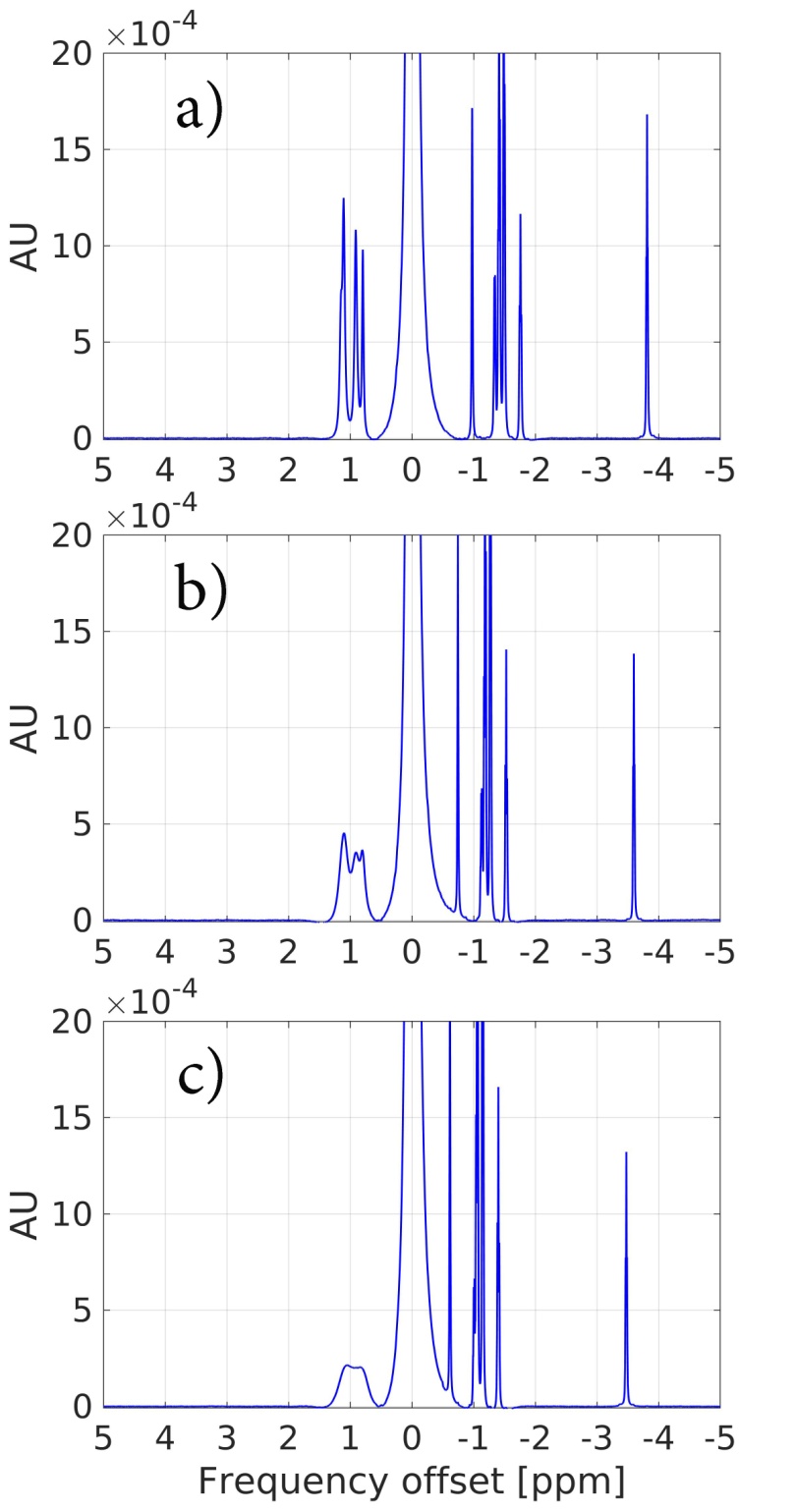


Fig. 11S. 1H-NMR spectrum of MI water solution (200 mM, pH=6.5) at: a) 4 C°, b) 25 C° and c) 37 C°.

**Creatine (100 mM)**


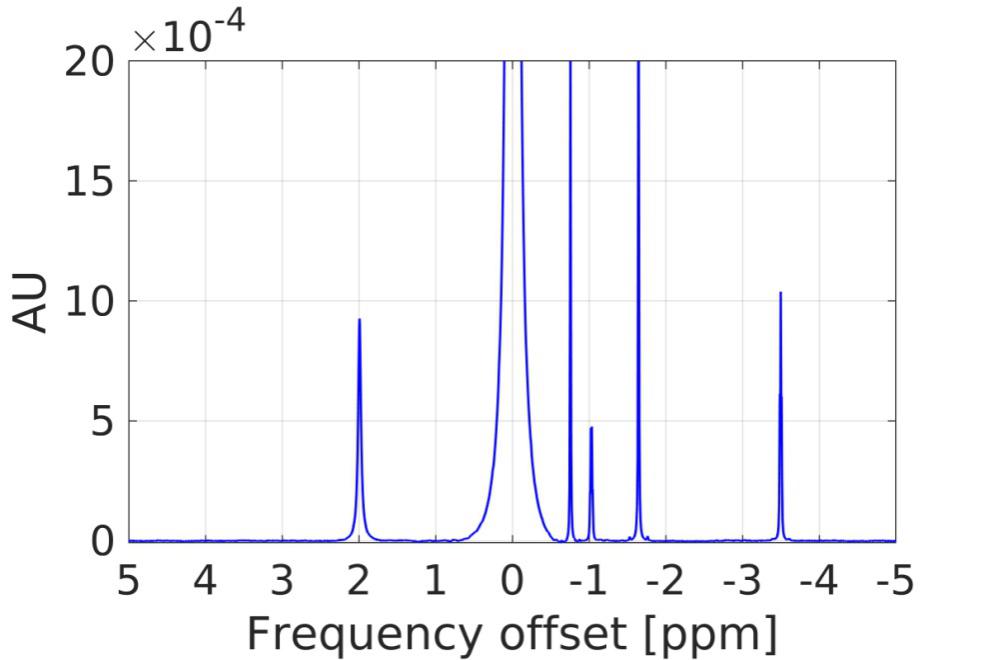


Fig. 12S. 1H-NMR spectrum of Cr water solution (100 mM, pH=5) at 37 C°.

**Phosphocreatine (100 mM)**


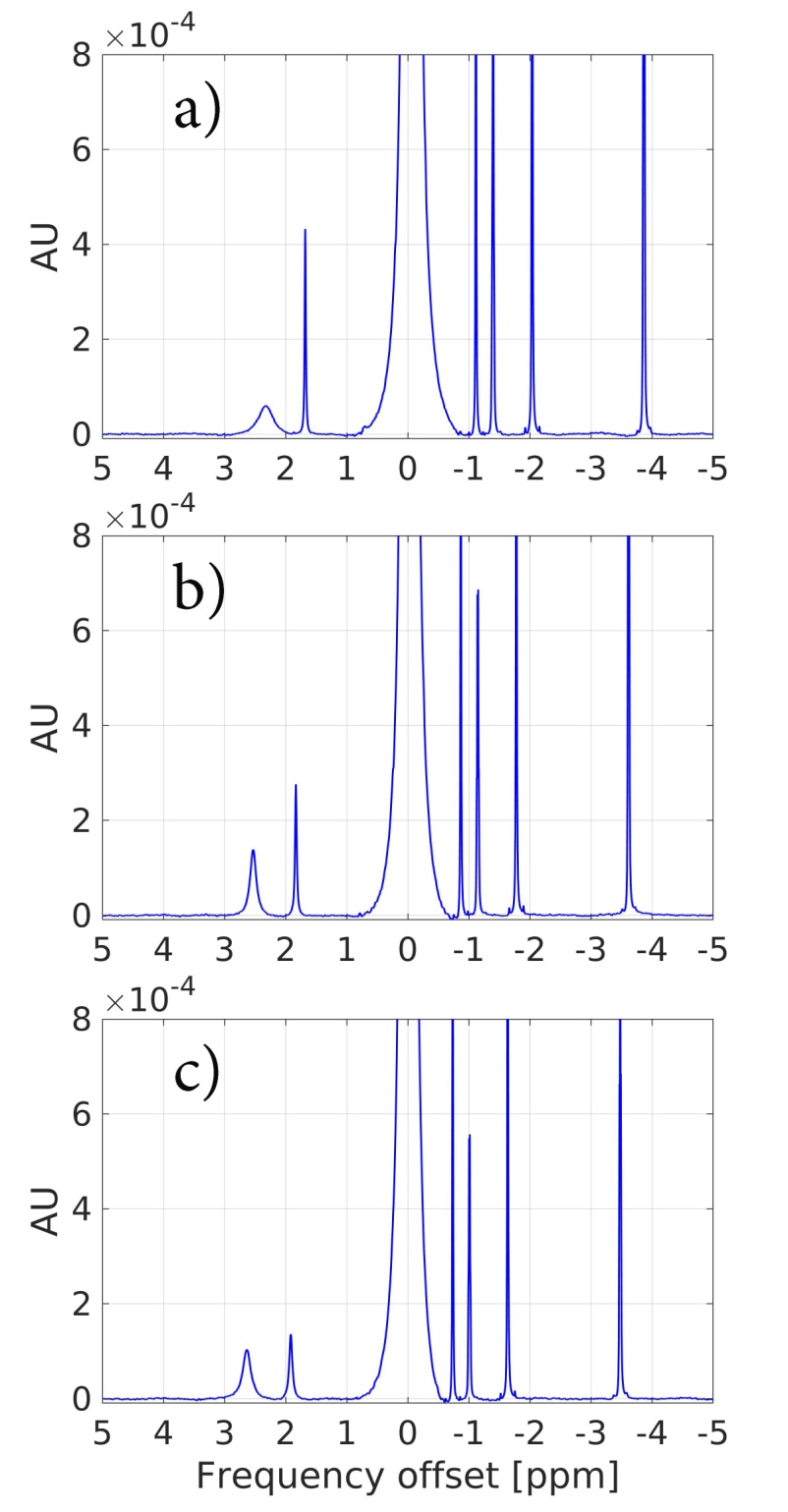


Fig. 13S. 1H-NMR spectrum of PCr water solution (100 mM, pH=7) at: a) 4 C°, b) 25 C° and c) 37 C°.

**ɣ-aminobutyric acid (100 mM)**


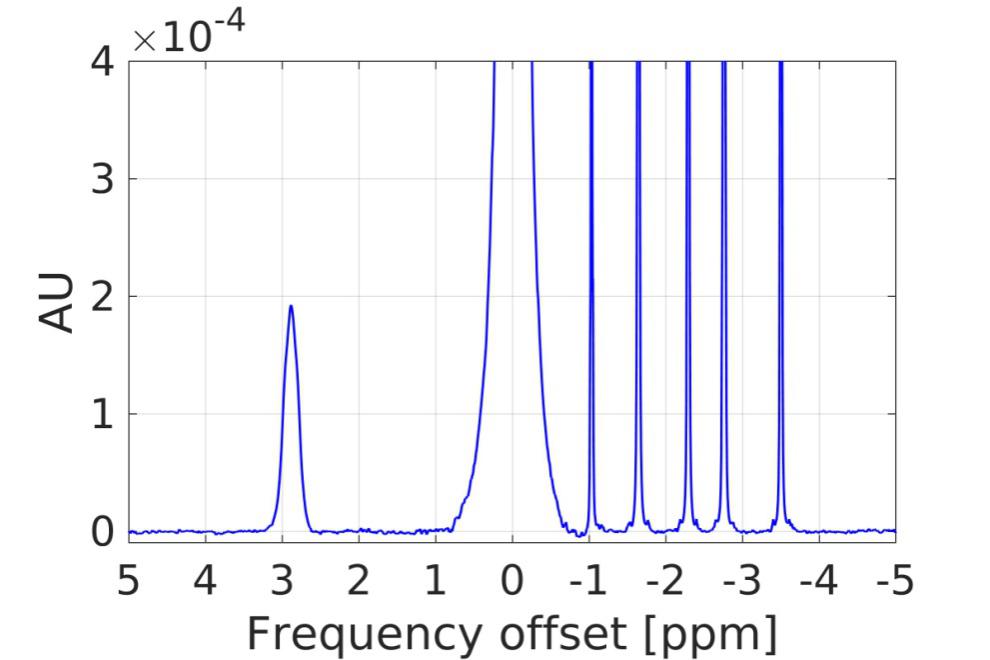


Fig. 14S. 1H-NMR spectrum of GABA water solution (100 mM, pH=4) at 37 C°.

**Taurine (200 mM)**


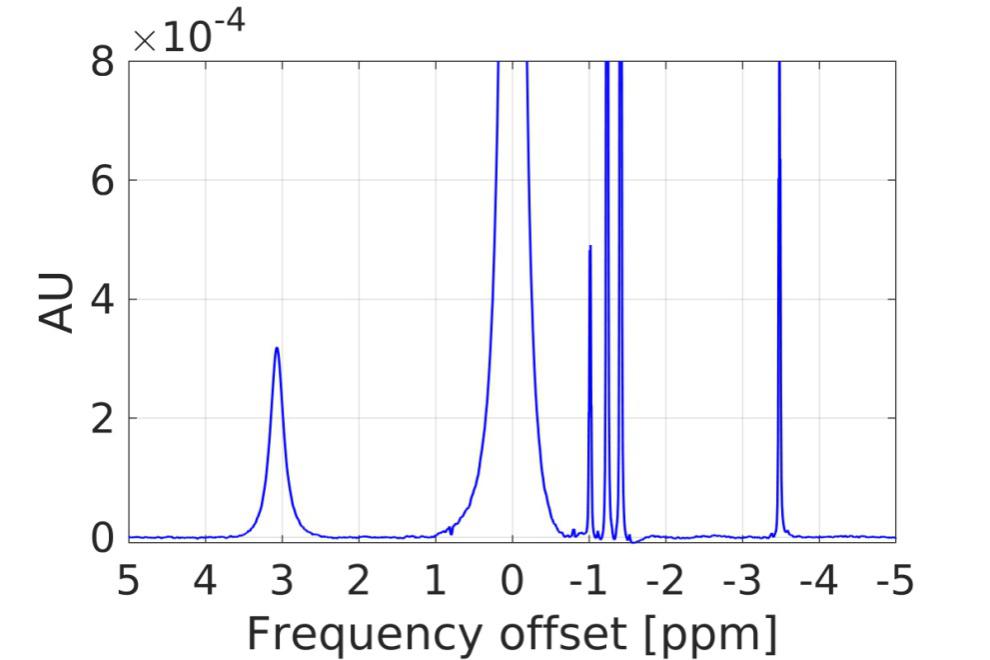


Fig. 15S. 1H-NMR spectrum of Tau water solution (200 mM, pH=4) at 37 C°.

**Glutamate (50 mM)**


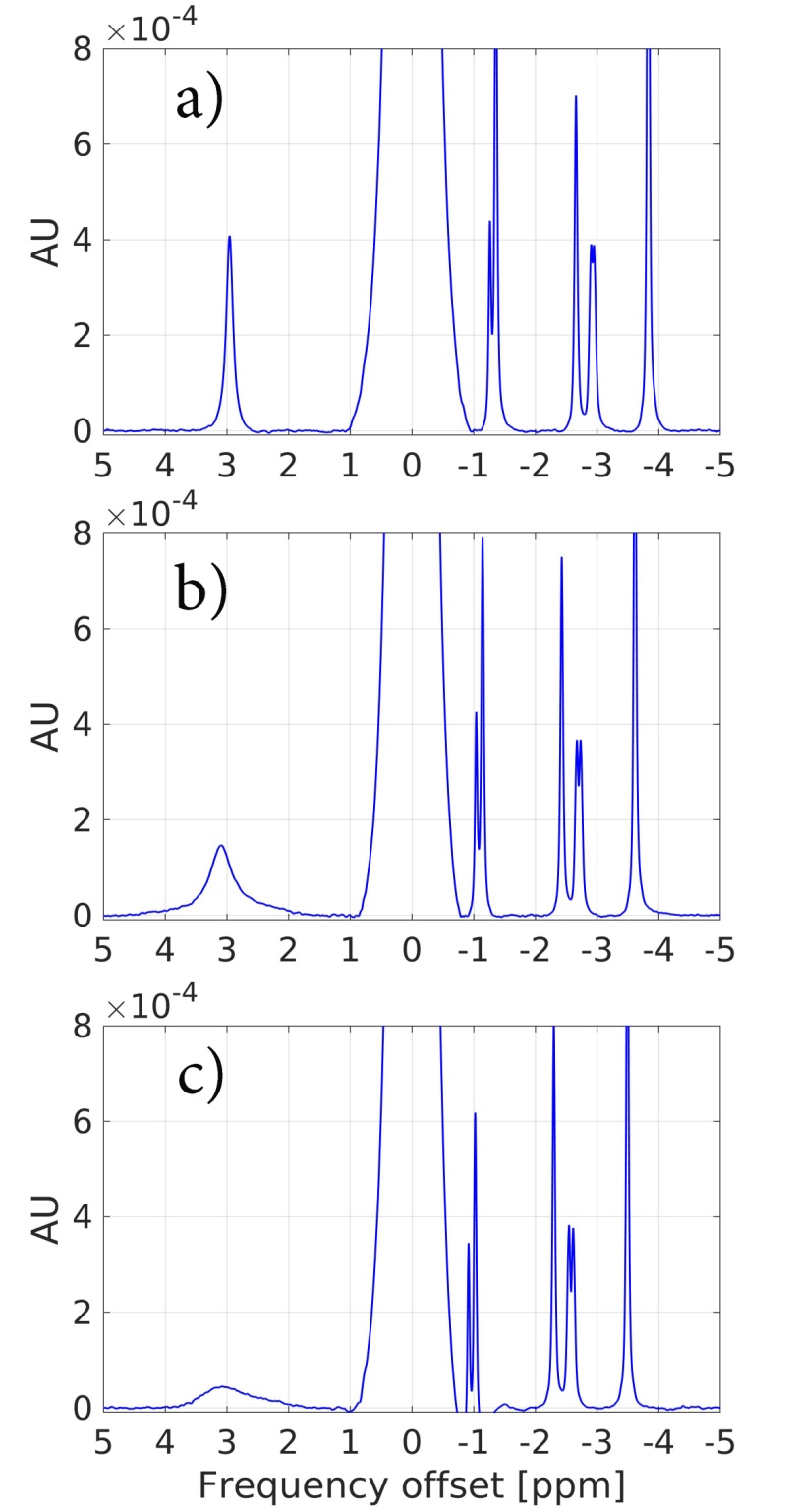


Fig. 16S. 1H-NMR spectrum of Glu water solution (50 mM, pH=5) at: a) 4 C°, b) 25 C° and c) 37 C°.

**Glutamine (100 mM)**


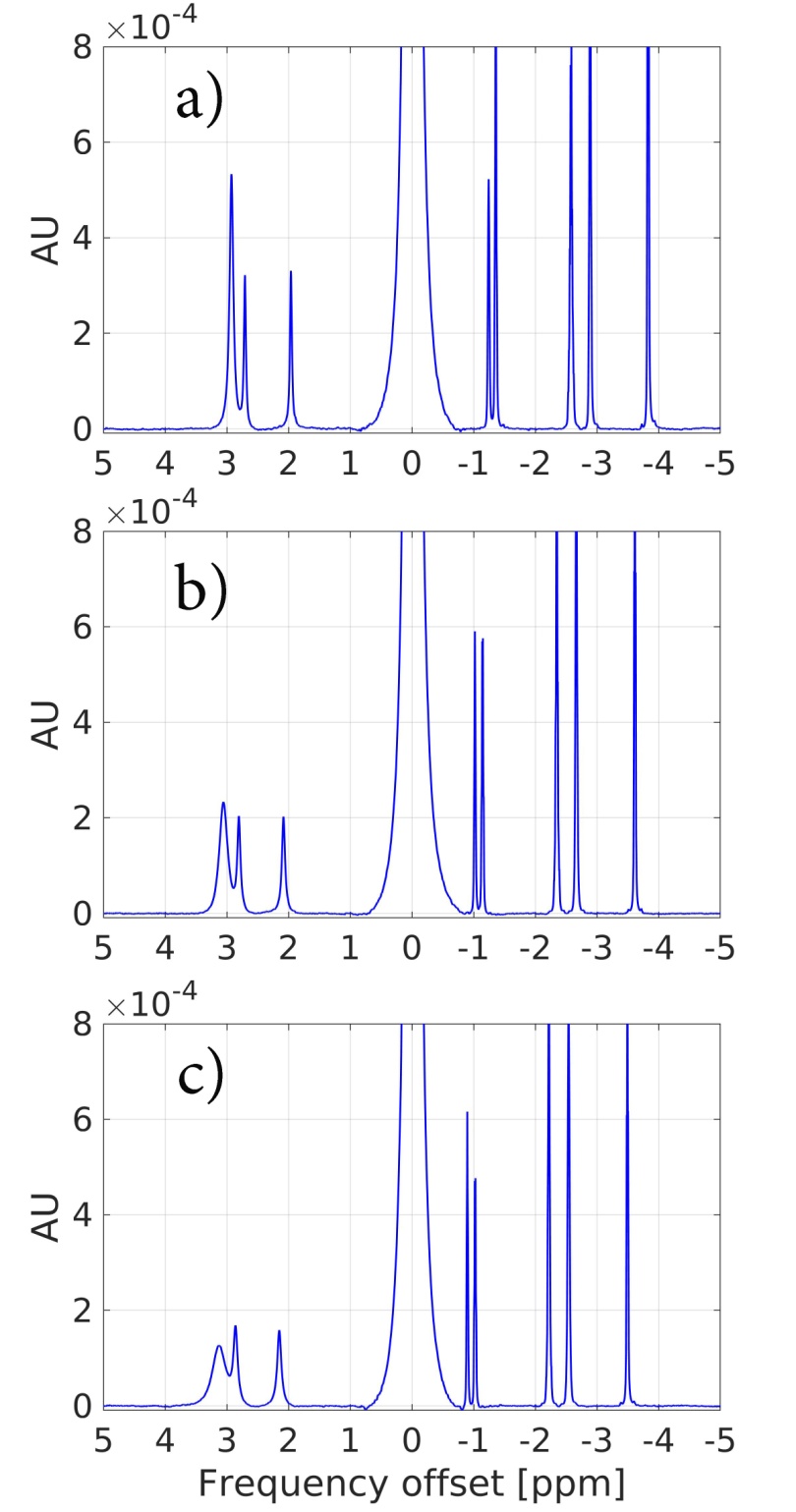


Fig. 17S. 1H-NMR spectrum of Gln water solution (100 mM, pH=4) at: a) 4 C°, b) 25 C° and c) 37 C°.

**N-acetyl-aspartate (100 mM)**


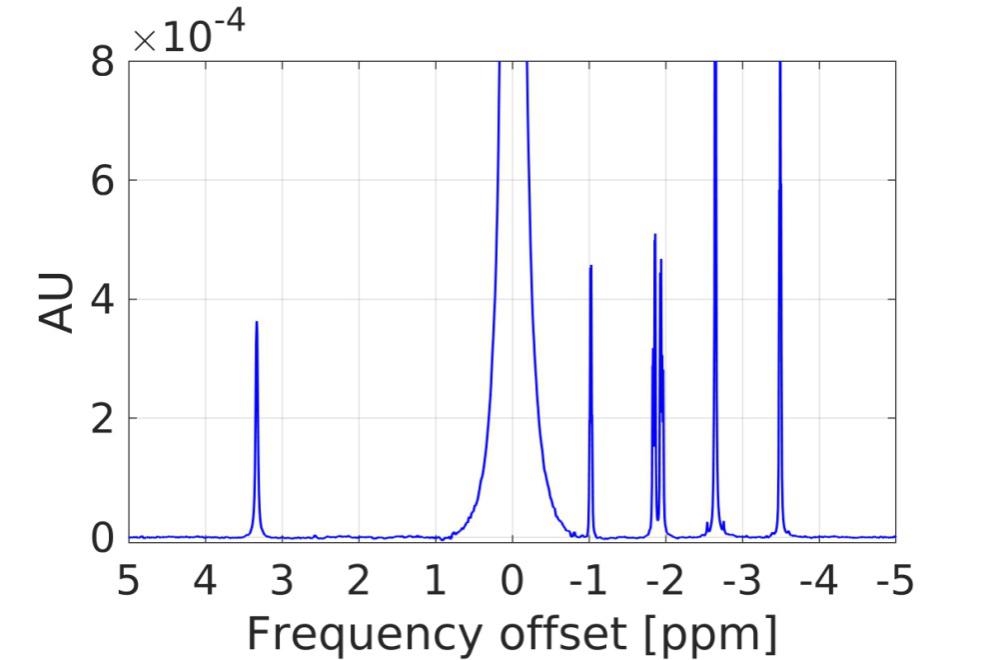


Fig. 18S. 1H-NMR spectrum of NAA water solution (100 mM, pH=7) at 37 C°.

**Field strength dependence of water T1 and T2 relaxation times**

Water T1 and T2 values were pooled from multiple sources^[[1]](#endnote-1),^^[[2]](#endnote-2),^^[[3]](#endnote-3),^^[[4]](#endnote-4),^^[[5]](#endnote-5)^. T1 and T2 relaxation times at 14.1T were obtained by extrapolation using a linear and an exponential fit, respectively, to the literature values in the range 3T-11.7T.

**
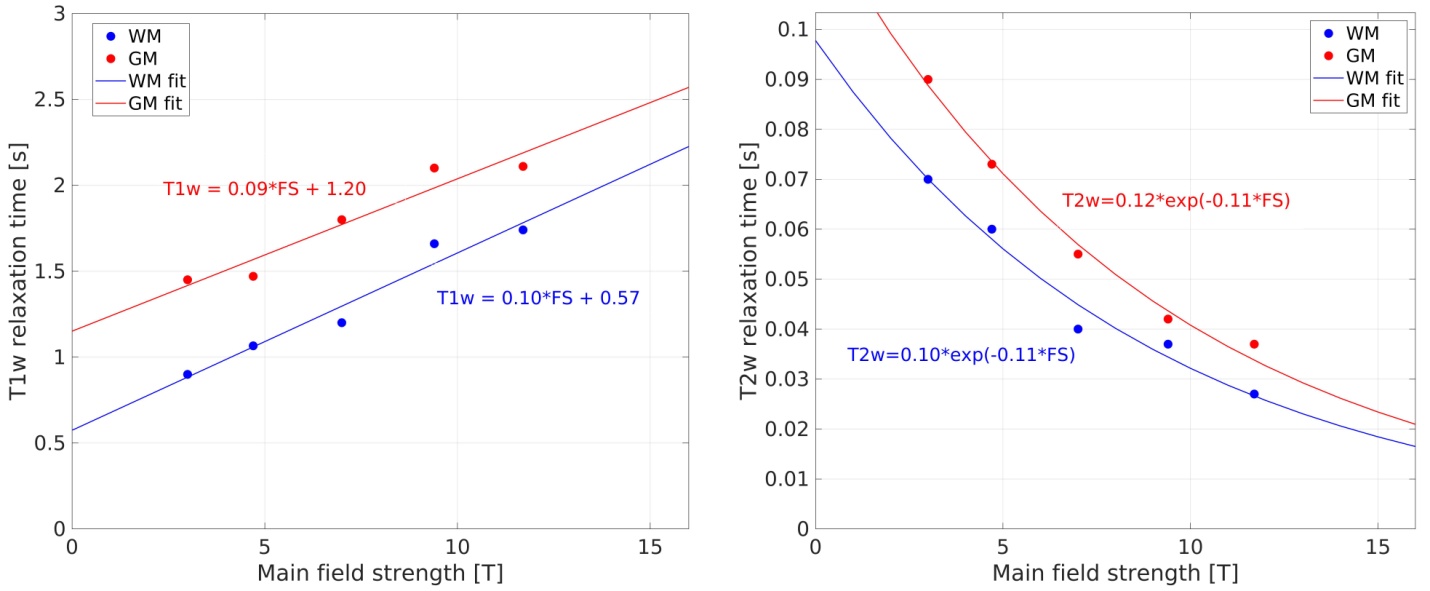
**

Fig. 19S. Field strength dependence of water T1 (left) and water T2 (right) relaxation times.

**Optimization of CEST prepulse parameters at different field strenths**

**BME simulations at 3T**

**
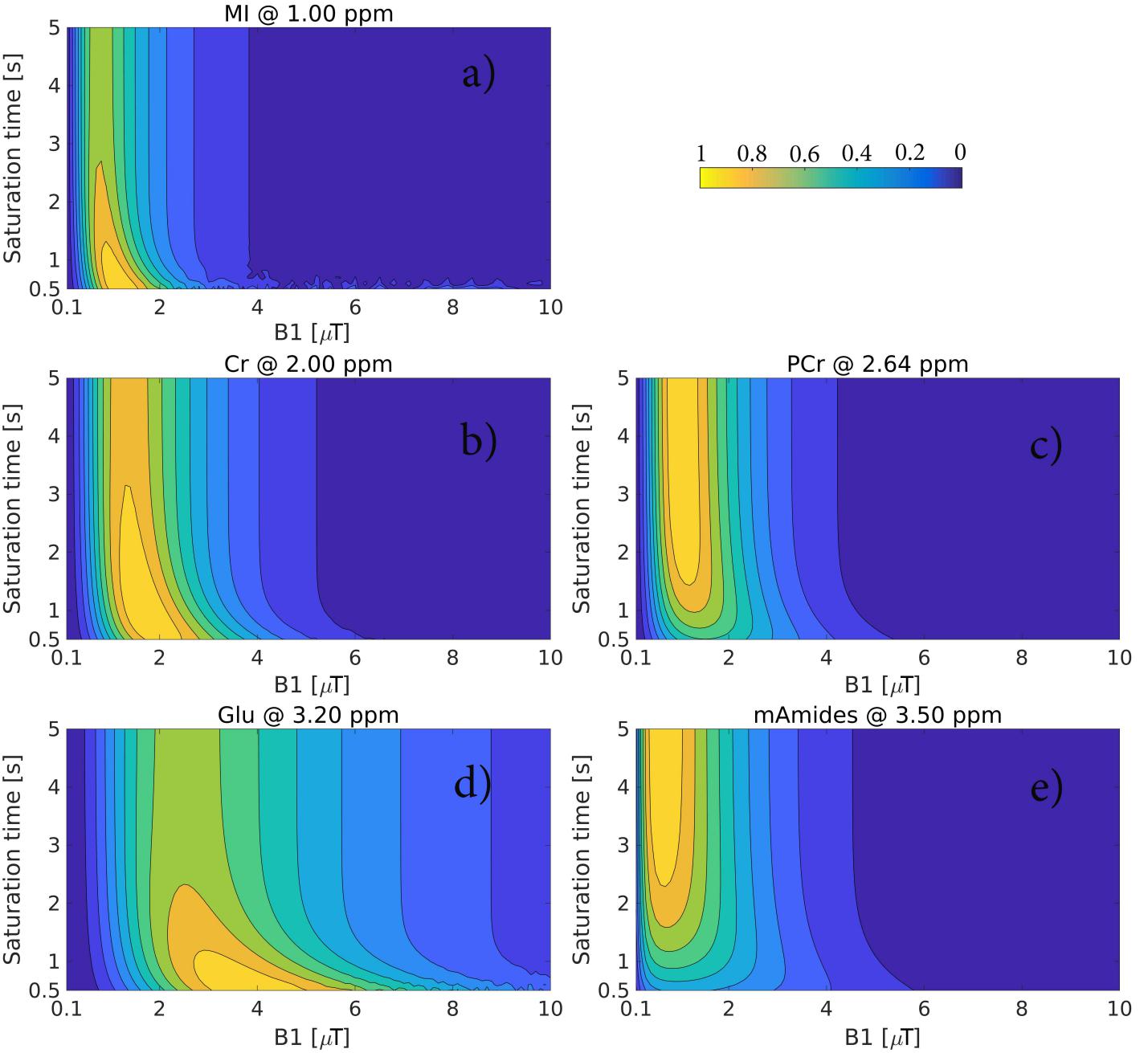
**

Fig. 20S. BME-simulated normalized effect size (pH=7, 3T field strength) for the following metabolites at their resonance frequencies: (a) MI (at 1.00 ppm), (b) Cr (at 2.00 ppm), (c) PCr (at 2.64 ppm), (d) Glu (at 3.20 ppm) and (e) mAmides (at 3.50 ppm) as a function of B1 amplitude and saturation time. Each map is an average of normalized (by maximum) maps simulated with WM and GM water T1 and T2 relaxation times. The contour plots overlaid on the maps delineate the regions with the effect size variation within 10%. See top right corner for color coding legend.

**BME simulations at 4.7T**

**
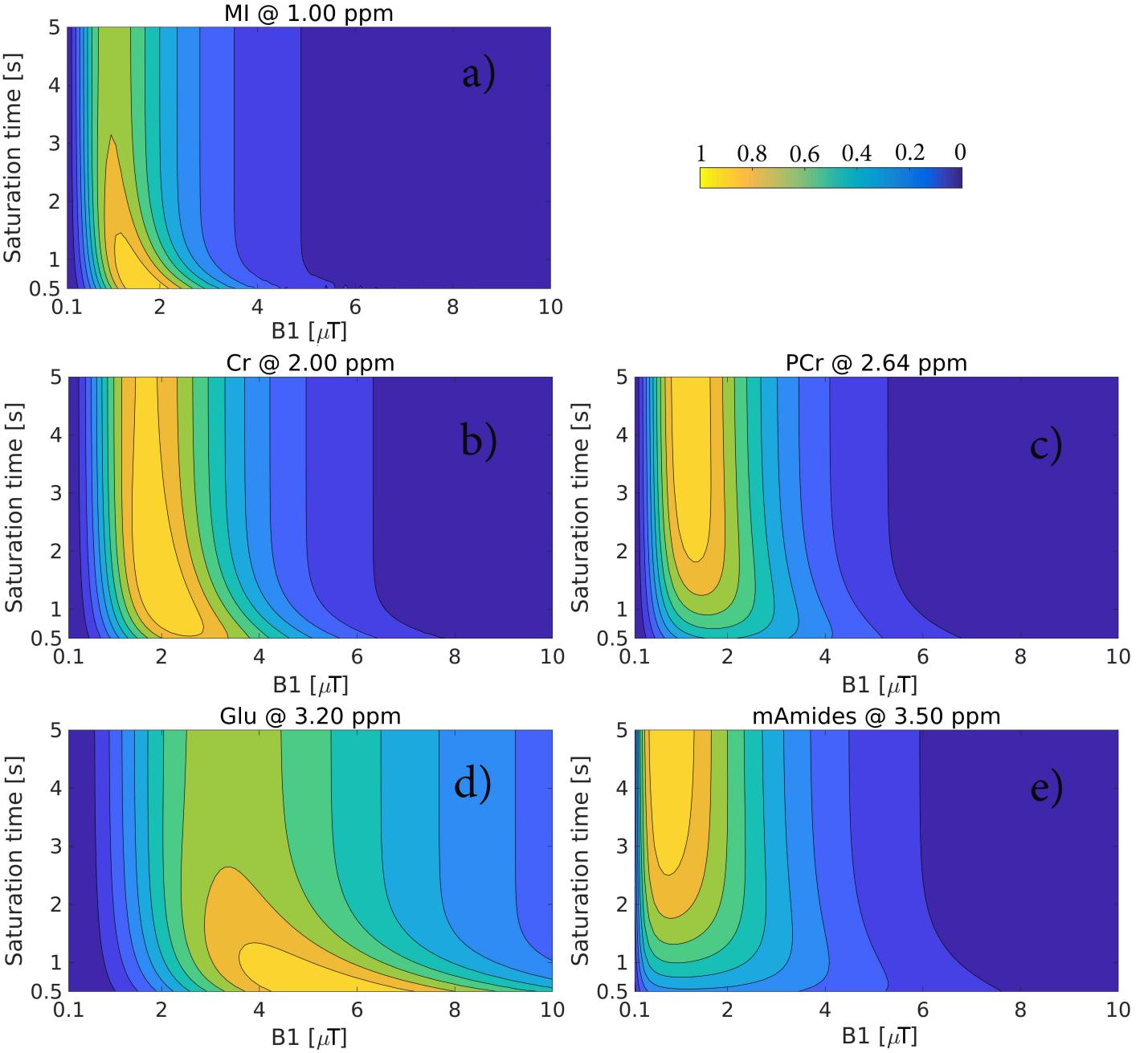
**

Fig. 21S. BME-simulated normalized effect size (pH=7, 4.7T field strength) for the following metabolites at their resonance frequencies: (a) MI (at 1.00 ppm), (b) Cr (at 2.00 ppm), (c) PCr (at 2.64 ppm), (d) Glu (at 3.20 ppm) and (e) mAmides (at 3.50 ppm) as a function of B1 amplitude and saturation time. Each map is an average of normalized (by maximum) maps simulated with WM and GM water T1 and T2 relaxation times. The contour plots overlaid on the maps delineate the regions with the effect size variation within 10%. See top right corner for color coding legend.

**BME simulations at 7T**

**
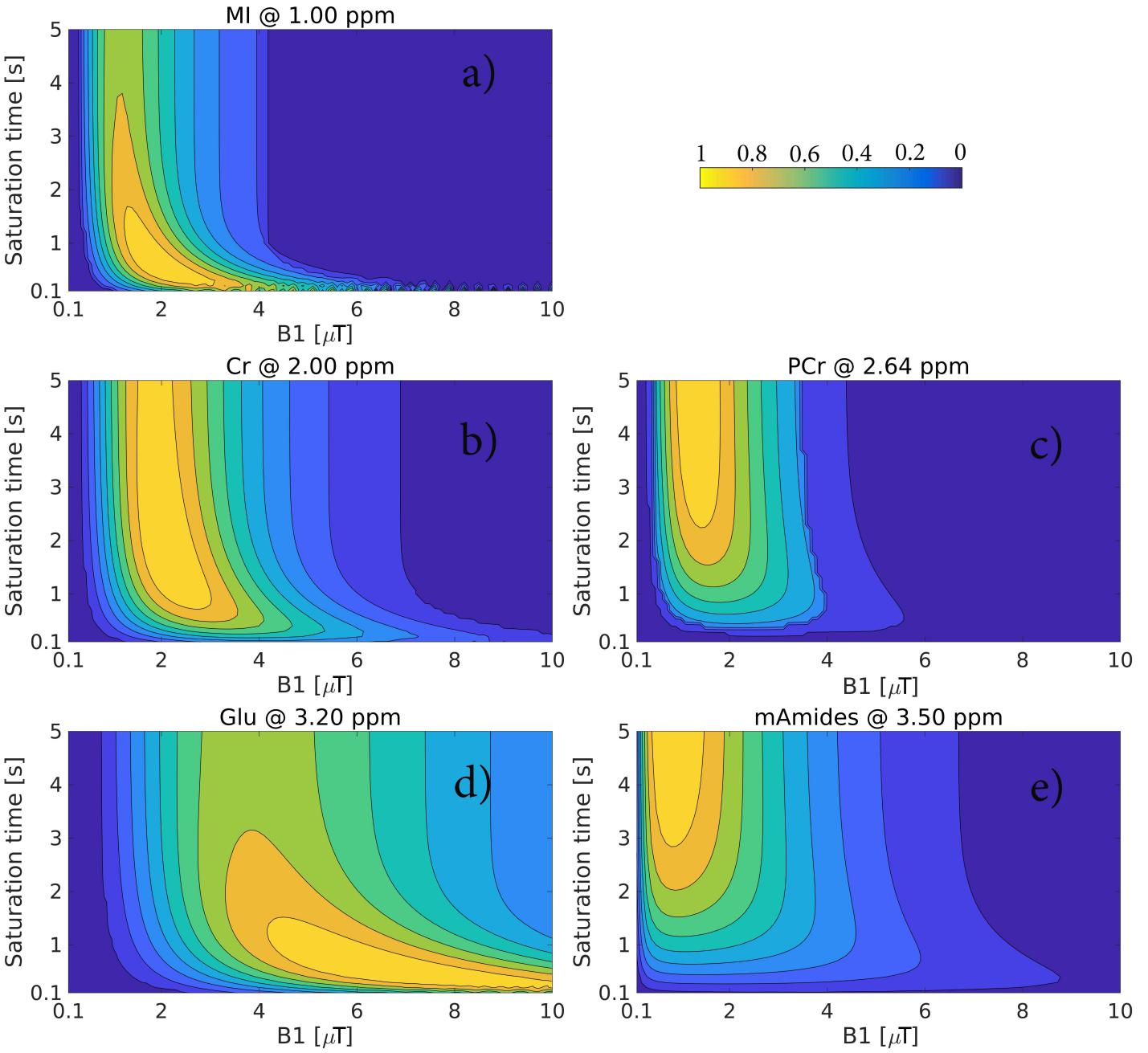
**

Fig. 22S. BME-simulated normalized effect size (pH=7, 7T field strength) for the following metabolites at their resonance frequencies: (a) MI (at 1.00 ppm), (b) Cr (at 2.00 ppm), (c) PCr (at 2.64 ppm), (d) Glu (at 3.20 ppm) and (e) mAmides (at 3.50 ppm) as a function of B1 amplitude and saturation time. Each map is an average of normalized (by maximum) maps simulated with WM and GM water T1 and T2 relaxation times. The contour plots overlaid on the maps delineate the regions with the effect size variation within 10%. See top right corner for color coding legend.

**BME simulations at 9.4T**

**
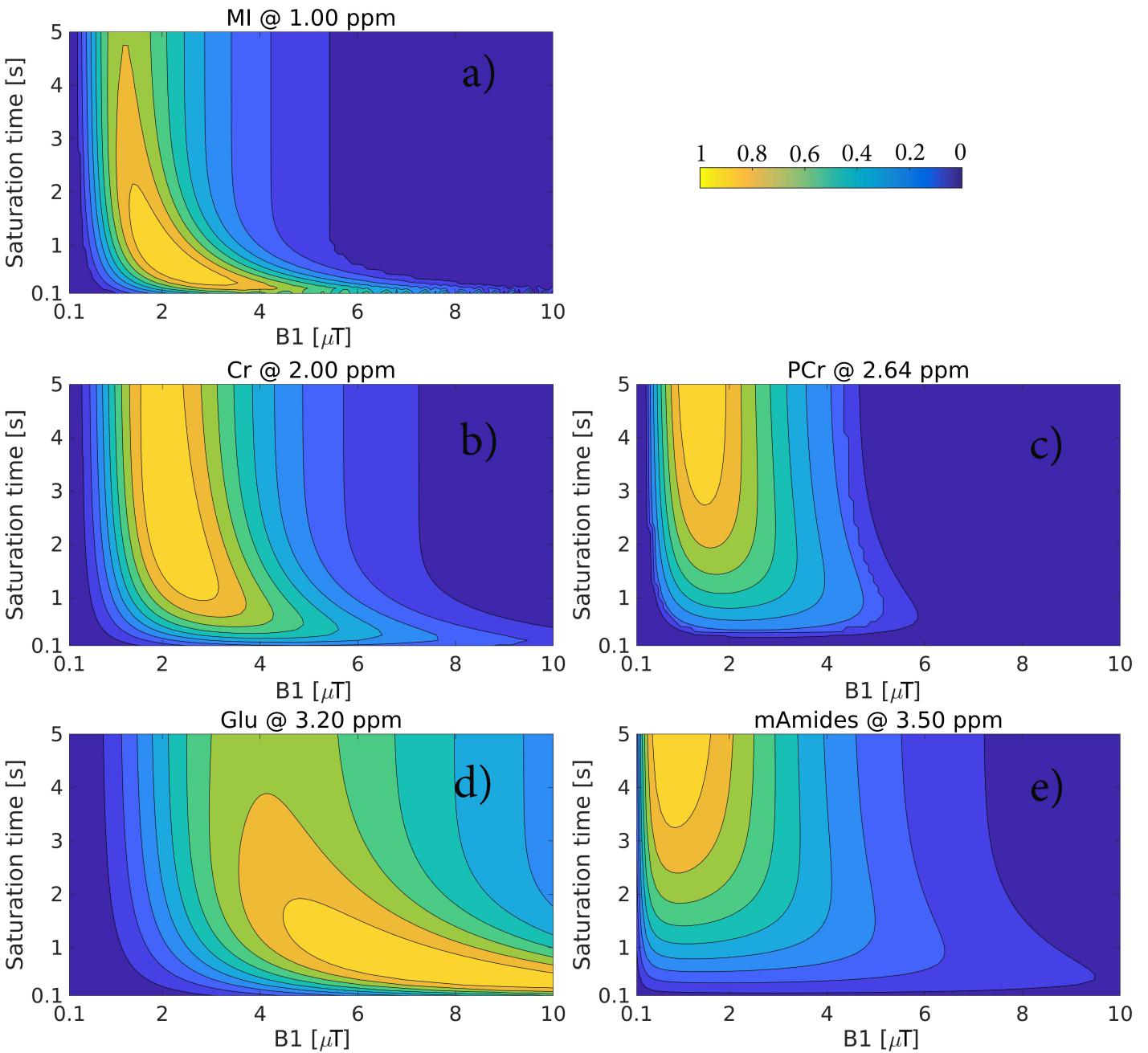
**

Fig. 23S. BME-simulated normalized effect size (pH=7, 9.4T field strength) for the following metabolites at their resonance frequencies: (a) MI (at 1.00 ppm), (b) Cr (at 2.00 ppm), (c) PCr (at 2.64 ppm), (d) Glu (at 3.20 ppm) and (e) mAmides (at 3.50 ppm) as a function of B1 amplitude and saturation time. Each map is an average of normalized (by maximum) maps simulated with WM and GM water T1 and T2 relaxation times. The contour plots overlaid on the maps delineate the regions with the effect size variation within 10%. See top right corner for color coding legend.

**BME simulations at 11.7T**

**
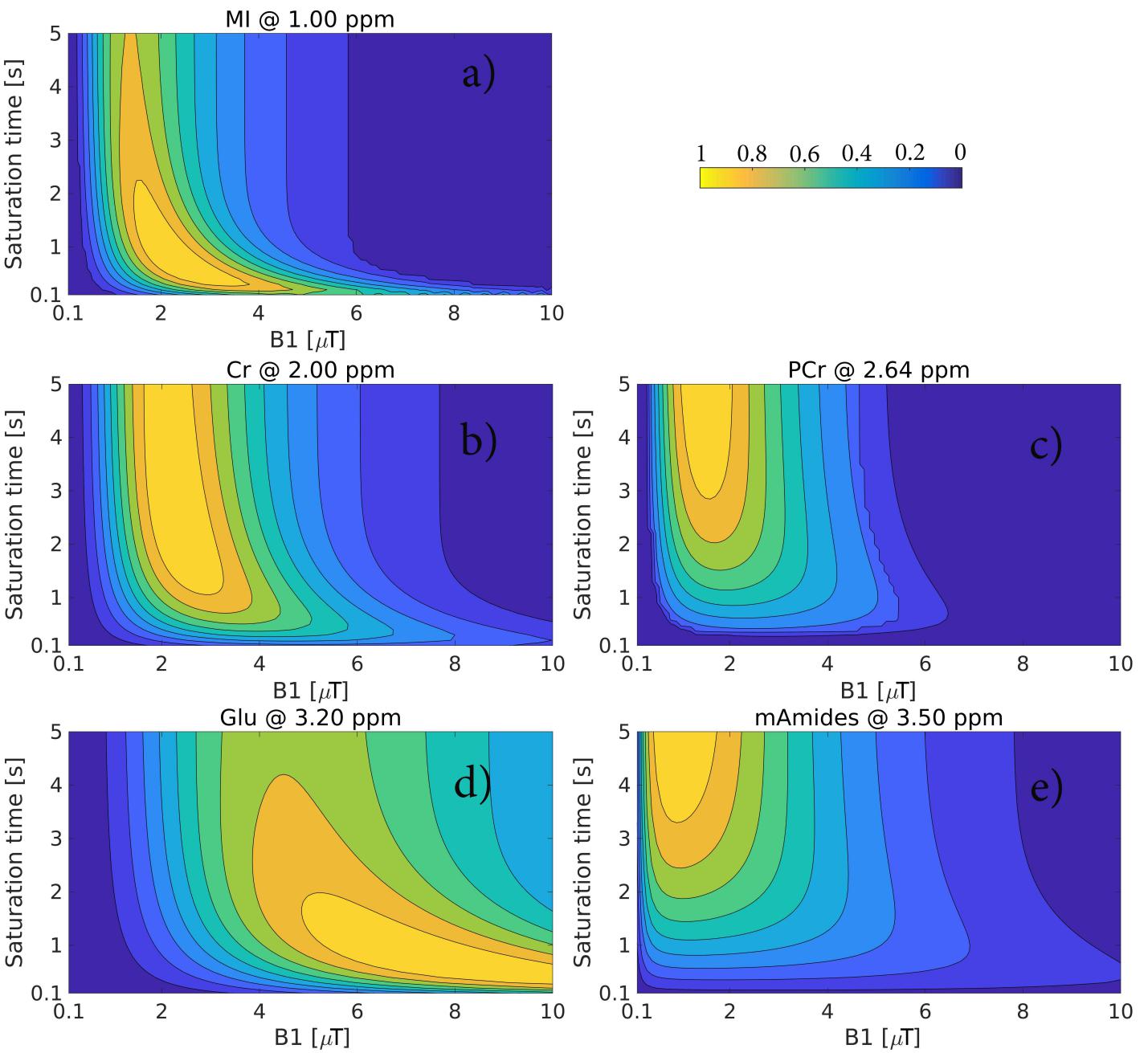
**

Fig. 24S. BME-simulated normalized effect size (pH=7, 11.7T field strength) for the following metabolites at their resonance frequencies: (a) MI (at 1.00 ppm), (b) Cr (at 2.00 ppm), (c) PCr (at 2.64 ppm), (d) Glu (at 3.20 ppm) and (e) mAmides (at 3.50 ppm) as a function of B1 amplitude and saturation time. Each map is an average of normalized (by maximum) maps simulated with WM and GM water T1 and T2 relaxation times. The contour plots overlaid on the maps delineate the regions with the effect size variation within 10%. See top right corner for color coding legend.

**BME simulations at 14.1T**

**
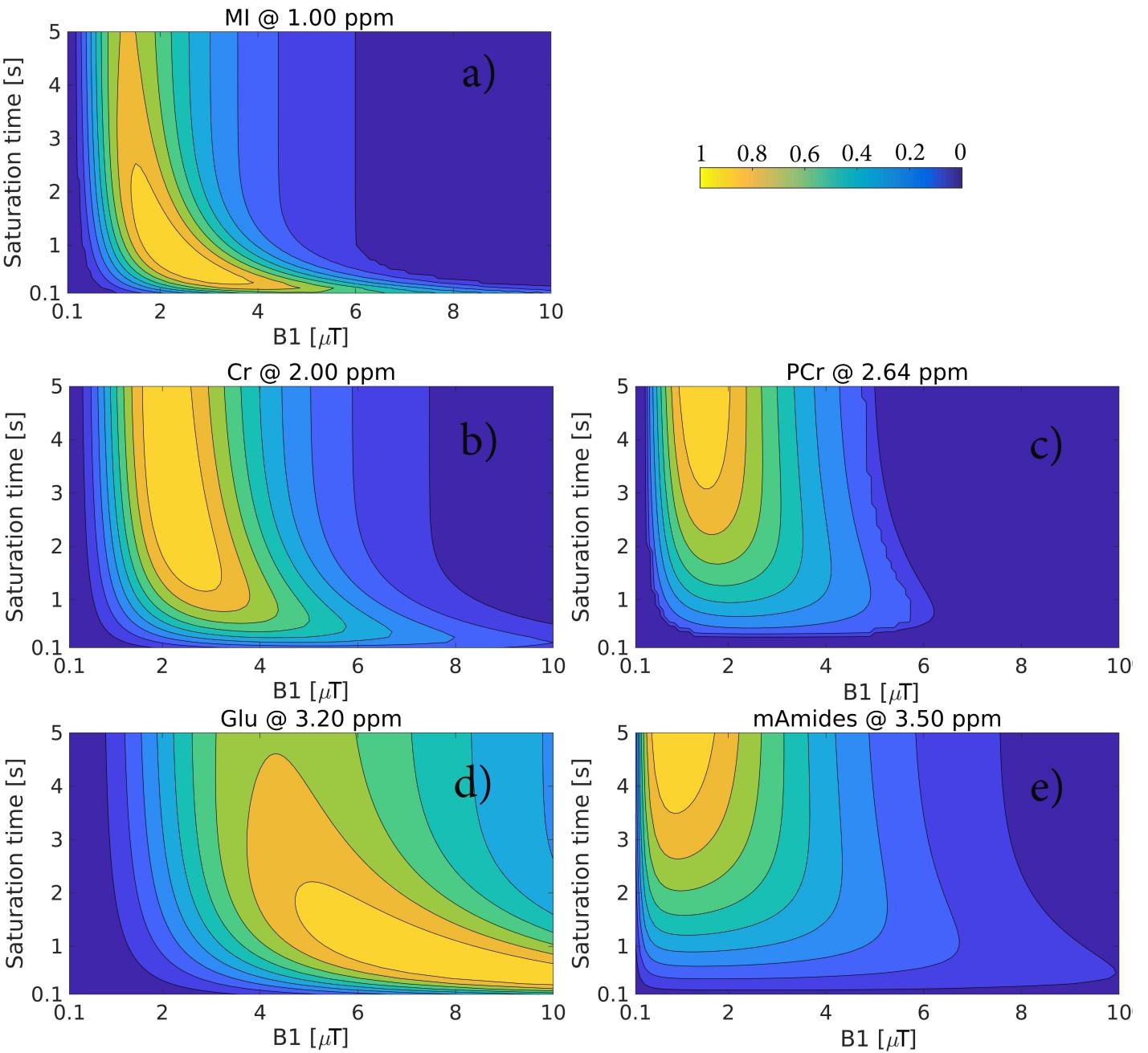
**

Fig. 25S. BME-simulated normalized effect size (pH=7, 14.1T field strength) for the following metabolites at their resonance frequencies: (a) MI (at 1.00 ppm), (b) Cr (at 2.00 ppm), (c) PCr (at 2.64 ppm), (d) Glu (at 3.20 ppm) and (e) mAmides (at 3.50 ppm) as a function of B1 amplitude and saturation time. Each map is an average of normalized (by maximum) maps simulated with WM and GM water T1 and T2 relaxation times. The contour plots overlaid on the maps delineate the regions with the effect size variation within 10%. See top right corner for color coding legend.

**CEST effects from glucose exchangeable protons**


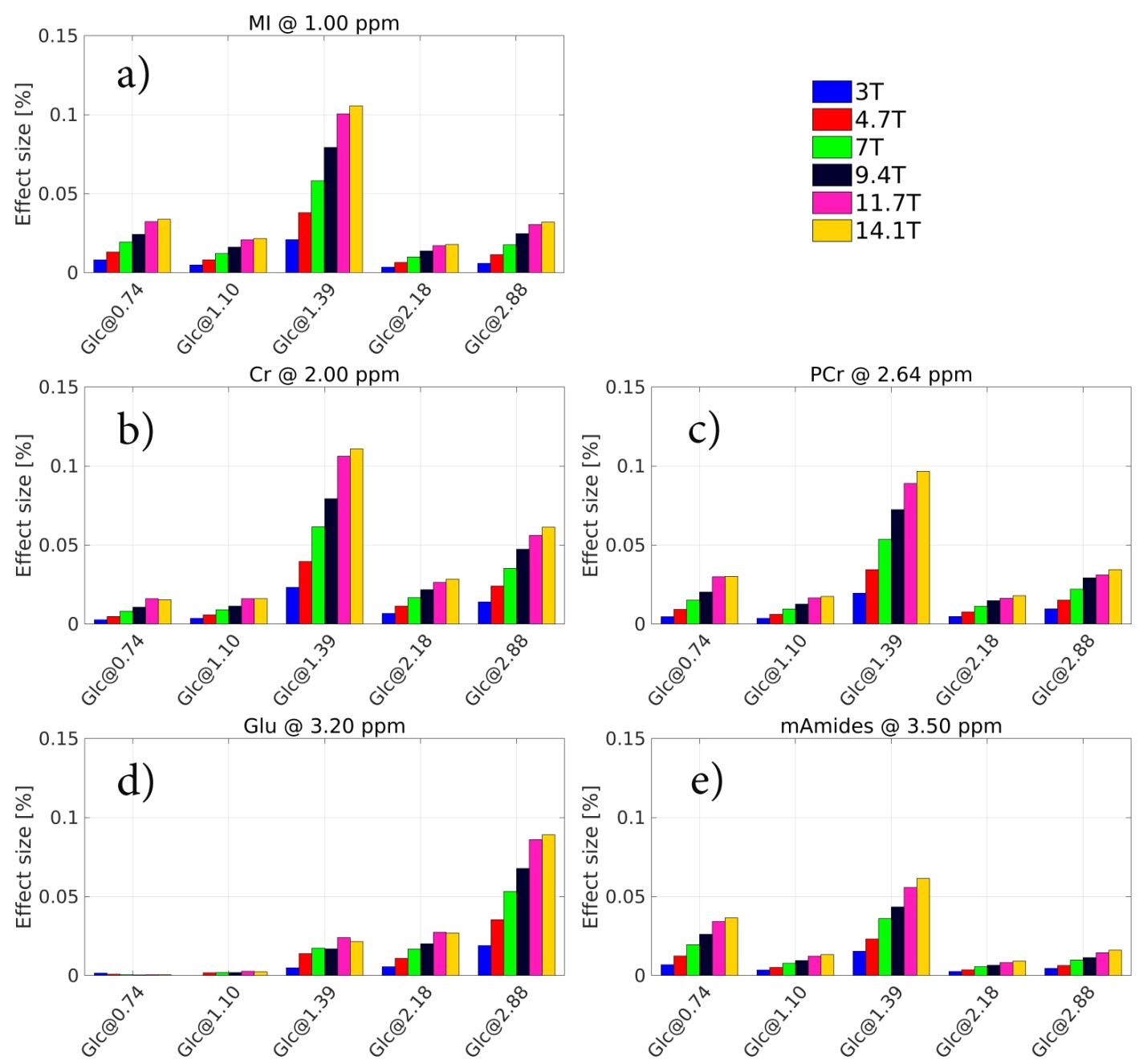


Fig. 26S. The comparison of BME-simulated CEST effects (pH=7) from Glc exchangeable protons in GM (see Table 1 in manuscript) at different field strengths. Shown are CEST effects from the individual exchangeable protons (X-axis). Each subplot was simulated at the optimum CEST parameters (see Table 2 in manuscript) for each particular metabolite at its resonance frequency: (a) MI (at 1.00 ppm), (b) Cr (at 2.00 ppm), (c) PCr (2.64 ppm), (d) Glu (3.20 ppm) and (e) mAmides (3.50 ppm). See top right corner for color coding legend.

**Optimization of CEST prepulse parameters for glucose at different field strengths**


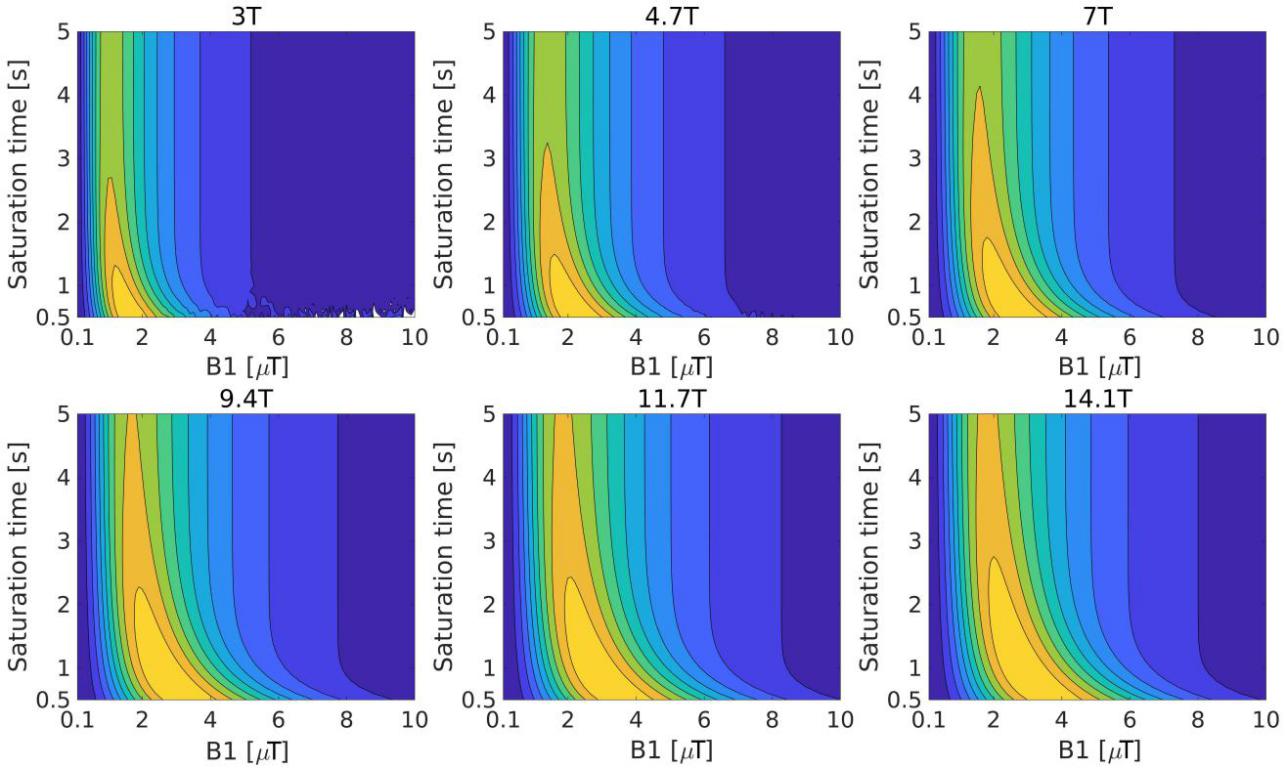


Fig. 27S. BME-simulated normalized effect size (pH=7) for Glc imaging (at a resonance frequencies of 1.39 ppm) as a function of B1 amplitude and saturation time. Each map is an average of normalized (by maximum) maps simulated with WM and GM water T1 and T2 relaxation times. The contour plots overlaid on the maps delineate the regions with the effect size variation within 10%. See top right corner for color coding legend.

**Literature**

1. Polders, D. L., Leemans, A., Luijten, P. R. & Hoogduin, H. Uncertainty estimations for quantitative in vivo MRI T1 mapping. J. Magn. Reson. **224,** 53–60 (2012). [↑](#endnote-ref-1)
2. Stanisz, G. J. et al. T1, T2 relaxation and magnetization transfer in tissue at 3T. Magn. Reson. Med. **54,** 507–512 (2005). [↑](#endnote-ref-2)
3. Bojorquez, J. Z. et al. What are normal relaxation times of tissues at 3 T? Magn. Reson. Imaging **35,** 69–80 (2017). [↑](#endnote-ref-3)
4. de Graaf, R. A. et al. High magnetic field water and metabolite proton T1 and T2 relaxation in rat brain in vivo. Magn. Reson. Med. **56,** 386–394 (2006). [↑](#endnote-ref-4)
5. Rooney, W. D. et al. Magnetic field and tissue dependencies of human brain longitudinal 1H2O relaxation in vivo. Magn. Reson. Med. **57,** 308–318 (2007). [↑](#endnote-ref-5)
